# Asymptotic Properties of Estimators for Seasonally Cointegrated State Space Models Obtained Using the CVA Subspace Method

**DOI:** 10.3390/e23040436

**Published:** 2021-04-08

**Authors:** Dietmar Bauer, Rainer Buschmeier

**Affiliations:** Department of Business Administration and Economics, Bielefeld University, Universitaetsstrasse 25, 33615 Bielefeld, Germany; RBuschmeier@uni-bielefeld.de

**Keywords:** cointegration, subspace algorithms, VARMA models, seasonality, C13, C32

## Abstract

This paper investigates the asymptotic properties of estimators obtained from the so called CVA (canonical variate analysis) subspace algorithm proposed by Larimore (1983) in the case when the data is generated using a minimal state space system containing unit roots at the seasonal frequencies such that the yearly difference is a stationary vector autoregressive moving average (VARMA) process. The empirically most important special cases of such data generating processes are the I(1) case as well as the case of seasonally integrated quarterly or monthly data. However, increasingly also datasets with a higher sampling rate such as hourly, daily or weekly observations are available, for example for electricity consumption. In these cases the vector error correction representation (VECM) of the vector autoregressive (VAR) model is not very helpful as it demands the parameterization of one matrix per seasonal unit root. Even for weekly series this amounts to 52 matrices using yearly periodicity, for hourly data this is prohibitive. For such processes estimation using quasi-maximum likelihood maximization is extremely hard since the Gaussian likelihood typically has many local maxima while the parameter space often is high-dimensional. Additionally estimating a large number of models to test hypotheses on the cointegrating rank at the various unit roots becomes practically impossible for weekly data, for example. This paper shows that in this setting CVA provides consistent estimators of the transfer function generating the data, making it a valuable initial estimator for subsequent quasi-likelihood maximization. Furthermore, the paper proposes new tests for the cointegrating rank at the seasonal frequencies, which are easy to compute and numerically robust, making the method suitable for automatic modeling. A simulation study demonstrates by example that for processes of moderate to large dimension the new tests may outperform traditional tests based on long VAR approximations in sample sizes typically found in quarterly macroeconomic data. Further simulations show that the unit root tests are robust with respect to different distributions for the innovations as well as with respect to GARCH-type conditional heteroskedasticity. Moreover, an application to Kaggle data on hourly electricity consumption by different American providers demonstrates the usefulness of the method for applications. Therefore the CVA algorithm provides a very useful initial guess for subsequent quasi maximum likelihood estimation and also delivers relevant information on the cointegrating ranks at the different unit root frequencies. It is thus a useful tool for example in (but not limited to) automatic modeling applications where a large number of time series involving a substantial number of variables need to be modelled in parallel.

## 1. Introduction

Many time series show seasonal patterns that, according to [1] for example, cannot be modeled appropriately using seasonal dummies because they exhibit a slowly trending behavior typical for unit root processes.

To model such processes in the vector autoregressive (VAR) framework, Ref. [2] (abbreviated as JS in the following) extend the error correction representation for seasonally integrated autoregressive processes pioneered by [3] to the multivariate case. This vector error correction formulation (VECM) models the yearly differences of a process observed *S* times per year. The model includes systems having unit roots at some or all of the possible locations $z_{j}=\exp\left(\frac{2\pi j}{S}i\right),j=0,...,S-1$ of seasonal unit roots. In JS all unit roots are assumed to be simple such that the process of yearly differences is stationary.

In this setting JS propose an estimator for the autoregressive polynomial subject to restrictions on its rank (the so-called cointegrating rank) at the unit roots $z_{j}$ based on an iterative scheme focusing on a pair of complex-conjugated unit roots (or the unit roots $z_{j}=1$ or $z_{j}=-1$ respectively) at a time. The main idea here is the reformulation of the model using the so called vector error correction representation. Beside estimators JS also derived likelihood ratio tests for the cointegrating rank at the various unit roots.

Refs. [4,5] propose simpler estimation schemes based on complex reduced rank regression (cRRR in the following). They also show that their numerically simpler algorithm leads to test statistics for the cointegrating rank that are asymptotically equivalent to the quasi maximum likelihood tests of JS. These schemes still typically alternate between cRRR problems corresponding to different unit roots until convergence, although a one step version estimating only once at each unit root exists. Ref. [6] provides updating equations for quasi maximum likelihood estimation in situations where constraints on the parameters prohibit focusing on one unit root at a time.

The leading case here is that of quarterly data (S=4) where potential unit roots are located at ±1 and ±i, implying that the VECM representation contains four potentially rank restricted matrices. However, increasingly time series of much higher sampling frequency such as hourly, daily or weekly observations are available. In such cases it is unrealistic that all unit roots are present. If a unit root is not present, the corresponding matrix in the VECM is of full rank. Therefore in situations with only a few unit roots being present, the VECM requires a large number of parameters to be estimated. Also in cases with a long period length (such as for example hourly data with yearly cycles) usage of the VECM involves the estimation of all coefficient matrices for lags for at least one year.

In general, for processes of moderate to large dimension the VAR framework involves estimation of a large number of parameters which potentially can be avoided by using the more flexible vector autoregressive moving average (VARMA) or the—in a sense—equivalent state space framework. This setting has been used in empirical research for the modeling of electricity markets, see the survey [7] for a long list of contributions. In particular, ref. [8] use the model described below without formal verification of the asymptotic theory for the quasi maximum likelihood estimation.

Recently, ref. [9] show that in the setting of dynamic factor models, typically used for observation processes of high dimension, the common assumption that the factors are generated using a vector autoregression jointly with the assumption that the idiosyncratic component is white noise (or more generally generated using a VAR or VARMA model independent of the factors) leads to a VARMA process. Also a number of papers (see for example [10,11,12]) show that in their empirical application the usage of VARMA models instead of approximations using the VAR model leads to superior prediction performance. This, jointly with the fact that the linearization of dynamic stochastic general equilibrium models (DSGE) leads to state space models, see e.g., [13], has fuelled recent interest in VARMA—and thus state space—modeling in particular in macroeconomics, see for example [14].

In this respect, quasi maximum likelihood estimation is the most often used approach for inference. Due to the typically highly non-convex nature of the quasi likelihood function (using the Gaussian density) in the VARMA setting, the criterion function shows many local maxima where the optimization can easily get stuck. Randomization alone does not solve the problem efficiently, as typically the parameter space is high-dimensional causing problems of the curse of dimensionality type.

Moreover, VARMA modeling requires a full specification of the state space unit root structure of the process, see [15]. The state space unit root structure specifies the number of common trends at each seasonal frequency (see below for definitions). For data of weekly or higher sampling frequency it is unlikely that the state space unit root structure is known prior to estimation. Testing all possible combinations is numerically infeasible in many cases.

As an attractive alternative in this respect the class of subspace algorithms is investigated in this paper. One particular member of this class, the so called canonical variate analysis (CVA) introduced by [16] (in the literature the algorithm is often called canonical correlation analysis; CCA), has been shown to provide system estimators which (under the assumption of known system order) are asymptotically equivalent to quasi maximum likelihood estimation (using the Gaussian likelihood) in the stationary case [17]. CVA shares a number of robustness properties in the stationary case with VAR estimators: [18] shows that CVA produces consistent estimators of the underlying transfer function in situations where the innovations are conditionally heteroskedastic processes of considerable generality. Ref. [19] shows that CVA provides consistent estimators of the transfer function even for stationary fractionally integrated processes, if the order of the system tends to infinity as a function of the sample size at a sufficient rate.

In the I(1) case [20] introduce a heuristic adaptation of the algorithm using the assumption of known cointegrating rank in order to show consistency for the corresponding transfer function estimators. However, the specification of the cointegrating rank is no easy task in itself. In case of misspecification of the cointegrating rank the properties of this approach are unclear. Ref. [21] states without proof that also the original CVA algorithm delivers consistent estimates in the I(1) case without the need to impose the true cointegrating rank.

Furthermore for I(1) processes [20] proposed various tests for the cointegrating rank and compared them to tests in the Johansen framework showing superior finite sample performance in particular for multivariate data sets with a large dimension of the modeled process.

This paper builds on these results and shows that CVA can also be used in the seasonally integrated case. The main contributions of the paper are:

(i) It is shown that the original CVA algorithm in the seasonally integrated case provides strongly consistent system estimators under the assumption of known system order (thus delivering the currently unpublished proof of the claim in the I(1) case in [21]).
(ii) Upper bounds for the order of convergence for the estimated system matrices are given, establishing the familiar superconsistency for the estimation of the cointegrating spaces at all unit roots.
(iii) Several tests for separate (that is for each unit root irrespective of the specification at the other potential unit roots) determination of the seasonal cointegrating ranks are proposed which are based on the estimated systems and are simple to implement.

The derivation of the asymptotic properties of the estimators is complemented by a simulation study and an application, both demonstrating the potential of CVA and one of the suggested tests. Jointly our results imply that CVA constitutes a very reasonable initial estimate for subsequent quasi likelihood maximization in the VARMA case. Moreover the method provides valuable information on the number of unit roots present in the process, which can be used for subsequent investigation at the very least by providing upper bounds on the number of common trends present at each unit root frequency. Contrary to the JS approach in the VAR framework these tests can be performed in parallel for all unit roots, eliminating the interdependence of the results inherent in the VECM representation. Moreover, they do not use the VECM representation involving a large number of parameters in the case of high sampling rates.

These properties make CVA a useful tool in automatic modeling of multivariate (with a substantial number of variables) seasonally (co-)integrated processes.

The paper is organized as follows: in the next section the model set and the main assumptions of the paper are presented. The estimation methods are described in Section 3. Section 4 states the consistency results. Inference on the cointegrating ranks is proposed in Section 5. Data preprocessing is discussed in Section 6. The simulations are contained in Section 7, while Section 8 discusses an application to real world data. Section 9 concludes the paper. Appendix A contains supporting material, Appendix C provides the proofs of the main results of this paper, which are based on preliminary results presented in Appendix B.

Throughout the paper we will use the symbols $o(g_{T})$ and $O(g_{T})$ to denote orders of almost sure convergence where *T* denotes the sample size, i.e., $x_{T}=o\left(g_{T}\right)$ if $x_{T}/g_{T}\to 0$ almost surely and $x_{T}=O\left(g_{T}\right)$ if $x_{T}/g_{T}$ is bounded almost surely for large enough *T* (that is there exists a constant M<∞ such that $\lim\sup_{T\to \infty }x_{T}/g_{T}\leq M$ a.s.). Furthermore, $o_{P}\left(g_{T}\right),O_{P}\left(g_{T}\right)$ denote the corresponding in probability versions.

## 2. Model Set and Assumptions

In this paper state space processes ${\left(y_{t}\right)}_{t\in Z},y_{t}\in R^{s},$ are considered which are defined as the solutions to the following equations for given white noise ${\left(\varepsilon_{t}\right)}_{t\in Z},\varepsilon_{t}\in R^{s},E\varepsilon_{t}=0,E\varepsilon_{t}\varepsilon_{t}^{\prime }=\Omega >0$:

(1) $$\begin{array}{ccc} x_{t+1} & = & Ax_{t}+K\varepsilon_{t},\\ y_{t} & = & Cx_{t}+\varepsilon_{t}. \end{array}$$

Here $x_{t}\in R^{n}$ denotes the unobserved state and $A\in R^{n\times n},C\in R^{s\times n}$ and $K\in R^{n\times s}$ define the state space system typically written as the tuple (A,C,K).

In this paper we consider without restriction of generality only minimal state space systems in innovations representation. For a minimal system the integer *n* is called the order of the system. As is well known (cf. e.g., [22]) minimal systems are only identified up to the choice of the basis of the state space. Two minimal systems (A,C,K) and $\left(\tilde{A},\tilde{C},\tilde{K}\right)$ are observationally equivalent if and only if there exists a nonsingular matrix $T\in R^{n\times n}$ such that $A=T\tilde{A}T^{-1},C=\tilde{C}T^{-1},K=T\tilde{K}$. For two observationally equivalent systems the impulse response sequences $k_{0}=I_{s},k_{j+1}=CA^{j}K=\tilde{C}{\tilde{A}}^{j}\tilde{K},j=0,1,...$ coincide.

Ref. [15] shows that the structure of the Jordan normal form of the matrix *A* determines the properties (such as stationarity) of the solutions to (1) for t∈Z. Eigenvalues of *A* on the unit circle lead to unit root processes in the sense of [15] who also define a *state space unit root structure* indicating the location and multiplicity of unit roots. A process ${\left(y_{t}\right)}_{t\in Z}$ with state space unit root structure $\Omega_{S}=\left\{\left(0,\left(c_{0}\right)\right),\left(2\pi /S,\left(c_{1}\right)\right),...,\left(\pi ,\left(c_{S/2}\right)\right)\right\}$ for some even integer *S* is called multi frequency I(1) (in short MFI(1)). Even *S* is chosen because it simplifies the notation by implying that S/2 also is an integer and z=−1 is a seasonal unit root. By adjusting the notation appropriately all results hold true for odd *S* as well).

If, moreover, such a process is observed for *S* periods per year, it is called *seasonal MFI(1)*. In this case the canonical form in [15] takes the following form:

(2) $$\begin{array}{ccc} A & = & \mathrm{diag}(A_{0},A_{1},\cdots ,A_{S/2},A_{•}),\\ A_{0} & = & I_{c_{0}},\\ A_{j} & = & \left[\begin{array}{cc} \cos\left(\omega_{j}\right)I_{c_{j}} & \sin\left(\omega_{j}\right)I_{c_{j}}\\ -\sin\left(\omega_{j}\right)I_{c_{j}} & \cos\left(\omega_{j}\right)I_{c_{j}} \end{array}\right],\;0<j<S/2,\\ A_{S/2} & = & -I_{c_{S/2}},\\ C & = & \left[\begin{array}{cccccccccccc} C_{0,R} & | & C_{1,R} & C_{1,I} & \cdots & \cdots & C_{S/2-1,R} & C_{S/2-1,I} & | & C_{S/2} & | & C_{•} \end{array}\right]\\ \; & = & \left[\begin{array}{ccccccccc} C_{0} & | & C_{1} & \cdots & C_{S/2-1} & | & C_{S/2} & | & C_{•} \end{array}\right],\\ K & = & {\left[\begin{array}{cccccccccccc} K_{0,R}^{\prime } & | & K_{1,R}^{\prime } & K_{1,I}^{\prime }| & \cdots & \cdots & |K_{S/2-1,R}^{\prime } & K_{S/2-1,I}^{\prime } & | & K_{S/2}^{\prime } & | & K_{•}^{\prime } \end{array}\right]}^{\prime } \end{array}$$

where $\omega_{j}=2\pi j/S,j=0,\cdots ,S/2$ denote the unit root frequencies and $C_{j,R}\in R^{s\times c_{j}},C_{j,I}\in R^{s\times c_{j}},K_{j,R}\in R^{c_{j}\times s},K_{j,I}\in R^{c_{j}\times s}$ where $0\leq c_{j}\leq s,0\leq j\leq S/2$. Furthermore for $C_{j,C}:=C_{j,R}-iC_{j,I}$ it holds that $C_{j,C}^{\prime }C_{j,C}=I_{c_{j}}$ and $K_{j,C}=K_{j,R}+iK_{j,I}$ is of full row rank and positive upper triangular ($C_{0,I}=C_{S/2,I}=0,K_{0,I}=K_{S/2,I}=0$), see [15] for details. Finally $|\lambda_{max}\left(A_{•}\right)|<1$, where $\lambda_{max}\left(A\right)$ denotes an eigenvalue of the matrix A with maximal modulus. The stable subsystem $\left(A_{•},C_{•},K_{•}\right)$ is assumed to be in echelon canonical form (see [22]).

Using this notation the assumptions on the data generating process (dgp) in this paper can be stated as follows:

**Assumption 1.** *${\left(y_{t}\right)}_{t\in Z}$ has a minimal state space representation $\left(A_{\circ },C_{\circ },K_{\circ }\right),A_{\circ }\in R^{n\times n}$ of the form (2) with minimal $\left(A_{\circ ,•},C_{\circ ,•},K_{\circ ,•}\right),A_{\circ ,•}\in R^{n_{•}\times n_{•}}$ in echelon canonical form where $c=n-n_{•}>0$.*

*Furthermore the stability assumption $|\lambda_{max}\left(A_{\circ ,•}\right)|<1$ and the strict minimum-phase condition $\rho_{0}:=\left|\lambda_{max}\left(A_{\circ }-K_{\circ }C_{\circ }\right)\right|<1$ hold.*

*The state at time t=1 is given by $x_{1}={\left[x_{1,0}^{\prime },...,x_{1,S/2}^{\prime },x_{1,•}^{\prime }\right]}^{\prime }$ where $x_{1,j}\in R^{\delta_{j}c_{j}}$ (for $\delta_{j}=2,0<j<S/2$ and $\delta_{j}=1$ else) is deterministic and $x_{1,•}=\sum_{j=1}^{\infty }A_{\circ ,•}^{j-1}K_{\circ ,•}\varepsilon_{1-j}$ is such that ${\left(x_{t,•}\right)}_{t\in Z}$ is stationary.*

*The noise process ${\left(\varepsilon_{t}\right)}_{t\in Z}$ is assumed to be a strictly stationary ergodic martingale difference sequence with respect to the filtration $F_{t}$ with zero conditional mean $E(\varepsilon_{t}|F_{t-1})=0$, deterministic conditional variance $E(\varepsilon_{t}\varepsilon_{t}^{\prime }|F_{t-1})=\Omega >0$ and finite fourth moments.*

Due to the block diagonal form of *A* the state equations are in a convenient form such that partitioning the state vector accordingly as

(3) $$x_{t}=\left(\begin{array}{c} x_{t,0}\\ x_{t,1}\\ \vdots \\ x_{t,S/2}\\ x_{t,•} \end{array}\right),$$

the blocks ${\left(x_{t,j}\right)}_{t\in Z},x_{t,j}\in R^{\delta_{j}c_{j}}$ for $c_{j}>0$ are unit root processes with state space unit root structure $\left\{\left(\omega_{j},\left(c_{j}\right)\right)\right\}$. Finally ${\left(x_{t,•}\right)}_{t\in Z}$ is assumed to be stationary due to the assumptions on $x_{1,•}$. If ${\left({\tilde{y}}_{t}\right)}_{t\in N}$ denotes a different solution to the state space equations corresponding to ${\tilde{x}}_{1}$ then (for t>1)

$${\tilde{y}}_{t}-y_{t}=CA^{t-1}\left({\tilde{x}}_{1}-x_{1}\right)=\sum_{j=0}^{S/2}C_{j}A_{j}^{t-1}\left({\tilde{x}}_{1,j}-x_{1,j}\right)+C_{•}A_{•}^{t-1}\left({\tilde{x}}_{1,•}-x_{1,•}\right).$$

Note that $C_{j}A_{j}^{t-1}z_{12}=\cos\left(\omega_{j}t\right)z_{1}+\sin\left(\omega_{j}t\right)z_{2},0<j<S/2$ (for appropriate vectors $z_{12},z_{1},z_{2}$),

$$C_{0}A_{0}^{t-1}=C_{0},\;C_{S/2}A_{S/2}^{t-1}={\left(-1\right)}^{t-1}C_{S/2}.$$

Therefore the sum $\sum_{j=0}^{S/2}C_{j}A_{j}^{t-1}\left({\tilde{x}}_{1,j}-x_{1,j}\right)$ can be modeled using a constant and seasonal dummies. The term $C_{•}A_{•}^{t-1}\left({\tilde{x}}_{1,•}-x_{1,•}\right)$ tends to zero with an exponential rate as t→∞ and hence does not influence the asymptotics.

Assumption 1 implies that the yearly difference

$$\begin{array}{ccc} y_{t}-y_{t-S} & = & CA^{S}x_{t-S}+\varepsilon_{t}+\sum_{i=1}^{S}CA^{i-1}K\varepsilon_{t-i}-Cx_{t-S}-\varepsilon_{t-S}\\ & = & \left(CA^{S}-C\right)x_{t-S}+v_{t}=\left(C_{•}A_{•}^{S}-C_{•}\right)x_{t-S,•}+v_{t} \end{array}$$

is a stationary VARMA process where $v_{t}=\varepsilon_{t}+\sum_{i=1}^{S}CA^{i-1}K\varepsilon_{t-i}-\varepsilon_{t-S}$ since $A_{j}^{S}=I_{\delta_{j}c_{j}}$. Thus the process according to Assumption 1 is a unit root process in the sense of [15]. Note that we do not assume that all unit roots are contained such that the spectral density of the stationary process ${\left(y_{t}-y_{t-S}\right)}_{t\in Z}$ may contain zeros due to overdifferentiation and hence the process potentially is not stably invertible. The special form of $A_{0}$ implies that I(1) processes are a special case of our dgp while I(d),d>1,d∈N, processes are not allowed for.

## 3. Canonical Variate Analysis

The main idea of CVA is that, given the state, the system equations (1) are linear in the system matrices. Therefore, based on an estimate of the state sequence, the system can be estimated using least squares regression. The estimate of the state is based on the following equation (for details see for example [17]):

Let $Y_{t,f}^{+}:={\left[y_{t}^{\prime },y_{t+1}^{\prime },\cdots ,y_{t+f-1}^{\prime }\right]}^{\prime }$ denote the vector of stacked observations for some integer f≥n and let $E_{t,f}^{+}:={\left[\varepsilon_{t}^{\prime },\varepsilon_{t+1}^{\prime },\cdots ,\varepsilon_{t+f-1}^{\prime }\right]}^{\prime }$. Further define $Y_{t,p}^{-}:={\left[y_{t-1}^{\prime },\cdots ,y_{t-p}^{\prime }\right]}^{\prime }$. Then (for t>p)

(4) $$\begin{array}{ccc} Y_{t,f}^{+} & = & O_{f}x_{t}+E_{f}E_{t,f}^{+}=O_{f}K_{p}Y_{t,p}^{-}+O_{f}{\left(A_{\circ }-K_{\circ }C_{\circ }\right)}^{p}x_{t-p}+E_{f}E_{t,f}^{+}\\ \; & = & \beta_{1}Y_{t,p}^{-}+N_{t,f}^{+} \end{array}$$

where $K_{p}:=\left[K_{\circ },{\bar{A}}_{\circ }K_{\circ },{\bar{A_{\circ }}}^{2}K_{\circ },\cdots ,{\bar{A_{\circ }}}^{p-1}K_{\circ }\right]$ for $\bar{A_{\circ }}:=A_{\circ }-K_{\circ }C_{\circ }$ and $O_{f}:={\left[C_{\circ }^{\prime },A_{\circ }^{\prime }C_{\circ }^{\prime },\cdots ,{\left(A_{\circ }^{f-1}\right)}_{\circ }^{\prime }C_{\circ }^{\prime }\right]}^{\prime }$. The strict minimum-phase assumption implies ${\bar{A_{\circ }}}^{p}\to 0$ for p→∞.

Let $\langle a_{t},b_{t}\rangle :=T^{-1}\sum_{t=p+1}^{T-f+1}a_{t}b_{t}^{\prime }$ for sequences ${\left(a_{t}\right)}_{t\in N}$ and ${\left(b_{t}\right)}_{t\in N}$. Then an estimate of $\beta_{1}$ is obtained from the reduced rank regression (RRR) $Y_{t,f}^{+}=\beta_{1}Y_{t,p}^{-}+N_{t,f}^{+}$ under the rank constraint $rank(\beta_{1})=n$. This results in the estimate $\hat{O_{f}}\hat{K_{p}}:=\left[{\left(\hat{\Xi_{f}^{+}}\right)}^{-1}{\hat{U}}_{n}{\hat{S}}_{n}\right]\left[{\hat{V}}_{n}^{\prime }{\left(\hat{\Xi_{p}^{-}}\right)}^{-1}\right]$ of $\beta_{1}$ using the singular value decomposition (SVD)

$$\hat{\Xi_{f}^{+}}{\hat{\beta }}_{1}\hat{\Xi_{p}^{-}}=\hat{U}\hat{S}{\hat{V}}^{\prime }={\hat{U}}_{n}{\hat{S}}_{n}{\hat{V}}_{n}^{\prime }+{\hat{R}}_{n}.$$

Here ${\hat{\beta }}_{1}=\langle Y_{t,f}^{+},Y_{t,p}^{-}\rangle {\langle Y_{t,p}^{-},Y_{t,p}^{-}\rangle }^{-1}$ denotes the unrestricted least squares estimate of $\beta_{1}$ and

(5) $$\hat{\Xi_{f}^{+}}:={\langle Y_{t,f}^{+},Y_{t,f}^{+}\rangle }^{-1/2},\;\hat{\Xi_{p}^{-}}:={\langle Y_{t,p}^{-},Y_{t,p}^{-}\rangle }^{1/2}.$$

Here the symmetric matrix square root is used. The definition is, however, not of importance and other square roots such as Cholesky factors could be used. ${\hat{U}}_{n}\in R^{fs\times n}$ denotes the matrix whose columns are the left singular vectors to the *n* largest singular values which are the diagonal entries in ${\hat{S}}_{n}:=\mathrm{diag}\left({\hat{\sigma }}_{1},{\hat{\sigma }}_{2},\cdots ,{\hat{\sigma }}_{n}\right),{\hat{\sigma }}_{1}\geq \cdots \geq {\hat{\sigma }}_{n}>0$ and ${\hat{V}}_{n}\in R^{ps\times n}$ contains the corresponding right singular vectors as its columns. ${\hat{R}}_{n}$ denotes the approximation error.

The system estimate $\left(\hat{A},\hat{C},\hat{K}\right)$ is then obtained using the estimated state ${\hat{x}}_{t}:=\hat{K_{p}}Y_{t,p}^{-},t=p+1,\cdots ,T+1$ via regression in the system equations.

In the algorithm a specific decomposition of the rank *n* matrix $\hat{O_{f}}\hat{K_{p}}$ into the two factors $\hat{O_{f}}$ and $\hat{K_{p}}$ is given such that $\hat{K_{p}}\hat{\Xi_{p}^{-}}{\left(\hat{\Xi_{p}^{-}}\right)}^{\prime }{\hat{K_{p}}}^{\prime }=I_{n}$. It is obvious that every other decomposition of $\hat{O_{f}}\hat{K_{p}}$ produces an estimated state sequence in a different coordinate system, leading to a different observationally equivalent representation of the same transfer function estimator. Therefore, with respect to consistency of the transfer function estimator it is sufficient to show that there exists a factorization of $\hat{O_{f}}\hat{K_{p}}$ leading to convergent system matrix estimators $\left(\tilde{A},\tilde{C},\tilde{K}\right)$, even if this factorization cannot be used in actual computations, as it requires information not known at the time of estimation.

In order to generate a consistent initial guess for subsequent quasi likelihood optimization in the set of all state space systems corresponding to processes with state space unit root structure $\Omega_{S}:=\left\{\left(\omega_{0},\left(c_{0}\right)\right),...,\left(\omega_{S/2},\left(c_{S/2}\right)\right)\right\}$, however, we will derive a realizable (for known integers $c_{j}$ and matrices $E_{j}$ such that $E_{j}^{\prime }C_{\circ ,j,C}=I_{c_{j}}$) consistent system estimate. To this end note that consistency of the transfer function implies (see for example [23]) that the eigenvalues ${\tilde{\lambda }}_{l}$ of $\hat{A}$ converge (in a specific sense) to the eigenvalues $\lambda_{j}$ of $A_{\circ }$. Therefore transforming $\hat{A}$ into complex Jordan normal form (where $\hat{A}$ is almost surely diagonalizable), ordering the eigenvalues such that groups of eigenvalues ${\tilde{\lambda }}_{l}\left(j\right),l=1,...,c_{j},$ converging to $\lambda_{j}$ are grouped together, we obtain a realizable system $\left(\overset{ˇ}{A},\overset{ˇ}{C},\overset{ˇ}{K}\right)$ where the diagonal blocks of the block diagonal matrix $\overset{ˇ}{A}$ corresponding to the unit roots converge to a diagonal matrix with the eigenvalues $z_{j}$ on the diagonal:   

$${\overset{ˇ}{A}}_{j,C}=\left[\begin{array}{cccc} {\tilde{\lambda }}_{1}\left(j\right) & 0 & \cdots & 0\\ 0 & {\tilde{\lambda }}_{2}\left(j\right) & \ddots & \vdots \\ \vdots & \ddots & \ddots & 0\\ 0 & \cdots & 0 & {\tilde{\lambda }}_{c_{j}}\left(j\right) \end{array}\right]\to A_{j,C}=\left[\begin{array}{cccc} z_{j} & 0 & \cdots & 0\\ 0 & z_{j} & \ddots & \vdots \\ \vdots & \ddots & \ddots & 0\\ 0 & \cdots & 0 & z_{j} \end{array}\right].$$

Replacing ${\overset{ˇ}{A}}_{j,C}$ by the limit $A_{j,C}$ and transforming the estimates to the real Jordan normal form, we obtain estimates $\left(\overset{˘}{A},\overset{ˇ}{C},\overset{ˇ}{K}\right)$ corresponding to unit root processes with state space unit root structure $\Omega_{S}$.

Note, however, that this representation not necessarily converges as perturbation analysis only implies convergence of the eigenspaces. Therefore in the final step the estimate $\left(\overset{˘}{A},\overset{ˇ}{C},\overset{ˇ}{K}\right)$ is converted such that we obtain convergence of the system matrix estimates. In the class of observationally equivalent systems with the matrix

$${\overset{˘}{A}}_{C}=\mathrm{diag}\left(A_{0,C},A_{1,C},\bar{A_{1,C}}...,\bar{A_{S/2-1,C}},A_{S/2,C},{\overset{ˇ}{A}}_{•}\right),\;A_{j,C}=I_{c_{j}}z_{j},$$

block diagonal transformations of the form $T=\mathrm{diag}(T_{0},T_{1},\bar{T_{1}},...,T_{S/2},I)$ do not change the matrix ${\overset{˘}{A}}_{C}$. Here the basis of the stable subsystem can be chosen such that the corresponding transformed $\left({\overset{˘}{A}}_{•},{\overset{˘}{C}}_{•},{\overset{˘}{K}}_{•}\right)$ is uniquely defined using an overlapping echelon form (see [22], Section 2.6). The impact of such transformations on the blocks of *C* is given by ${\overset{ˇ}{C}}_{j,C}T_{j}^{-1}$. Therefore, if for each j=0,...,S/2 a matrix $E_{j}\in C^{s\times c_{j}}$ is known such that $E_{j}^{\prime }C_{\circ ,j,C}\in C^{c_{j}\times c_{j}}$ is nonsingular, the restriction $E_{j}^{\prime }{\overset{˘}{C}}_{j,C}=I_{c_{j}}$ picks a unique representative $\left(\overset{˘}{A},\overset{˘}{C},\overset{˘}{K}\right)$ of the class of systems observationally equivalent to $\left(\overset{˘}{A},\overset{ˇ}{C},\overset{ˇ}{K}\right)$.

Note that this estimate $\left(\overset{˘}{A},\overset{˘}{C},\overset{˘}{K}\right)$ is realizable if the integers $c_{j}$ (needed to identify the $c_{j}$ eigenvalues of $\hat{A}$ closest to $z_{j}$), the matrices $E_{j}$ (needed to fix a basis for $x_{t,j}$) and the index corresponding to the overlapping echelon form for the stable part are known. Furthermore, this estimate corresponds to a process with state space unit root structure $\Omega_{S}$ and hence can be used as a starting value for quasi likelihood maximization.

Finally in this section it should be noted that the estimate of the state ${\hat{x}}_{t}$ here mainly serves the purpose of obtaining an estimator for the state space system. Based on this estimate, Kalman filtering techniques can be used to obtain different estimates of the state sequence. The relation between these different estimates is unclear and so is their usage for inference. For this paper the state estimates ${\hat{x}}_{t}$ are only an intermediate step in the CVA algorithm.

## 4. Asymptotic Properties of the System Estimators

As follows from the last section, the central step in the CVA procedure is a RRR problem involving stationary and nonstationary components. The asymptotic properties of the solution to such RRR problems are derived in Theorem 3.2. of [24]. Using these results the following theorem can be proved (see Appendix C.1):

**Theorem** **1.** *Let the process ${\left(y_{t}\right)}_{t\in Z}$ be generated according to Assumption 1. Let $\left(\hat{A},\hat{C},\hat{K}\right)$ denote the* CVA *estimators of the system matrices using the assumption of correctly specified order n with f≥n not depending on the sample size and finite and $p=o({\left(\log T\right)}^{\bar{a}})$ for some real $0<\bar{a}<\infty ,p\geq -d\log T/\log\rho_{0}$ for some d>1 where $0<\rho_{0}=\left|\lambda_{max}\left(A_{\circ }-K_{\circ }C_{\circ }\right)\right|<1$.* *Let $\left(A_{\circ },C_{\circ },K_{\circ }\right)$ be in the form given in (2) where $\left(A_{\circ ,•},C_{\circ ,•},K_{\circ ,•}\right)$ is in echelon canonical form and for each j=0,...,S/2 there exists a row selector matrix $E_{j}\in R^{s\times c_{j}}$ such that $E_{j}^{\prime }C_{\circ ,j,C}$ is non-singular. Then for some integer a:*

*(I)*  *$\hat{C}{\hat{A}}^{j}\hat{K}-C_{\circ }A_{\circ }^{j}K_{\circ }=O_{P}\left({\left(\log T\right)}^{a}/\sqrt{T}\right)$ for each j≥0.*
*(II)*  *Using $D_{x}=diag\left(T^{-1}I_{c},T^{-1/2}I_{n-c}\right)$ where $c=\sum_{j=0}^{S/2}c_{j}\delta_{j}$ we have*
  $$\left(\overset{˘}{A}-A_{\circ }\right)D_{x}^{-1}=O_{P}\left({\left(\log T\right)}^{a}\right),\sqrt{T}\left(\overset{˘}{K}-K_{\circ }\right)=O_{P}\left({\left(\log T\right)}^{a}\right),\left(\overset{˘}{C}-C_{\circ }\right)D_{x}^{-1}=O_{P}\left({\left(\log T\right)}^{a}\right)$$
*for some integer a<∞.*
*(III)*  *If the noise is assumed to be an iid sequence, then results (I) and (II) hold almost surely.*

Beside stating consistency in the seasonal integration case, the theorem also improves on the results of [20] in the I(1) case by showing that no adaptation of CVA is needed in order to obtain consistent estimators of the impulse response sequence or the system matrices. Note that this consistency result for the impulse response sequence concerns both the short and the long-run dynamics. In particular it implies that short-run prediction coefficients are consistent. Moreover the theorem establishes strong consistency rather than weak consistency as opposed to [20]. (II) establishes orders of convergence which, however, apply only to a transformed system that requires knowledge of the integers $c_{j}$ and matrices $E_{j}$ to be realized. No tight bounds for the integer *a* are derived, since they do not seem to be of much value.

Note that the assumptions on the innovations rule out conditionally heteroskedastic processes. However, since the proof mostly relies on convergence properties for covariance estimators for stationary processes and continuous mapping theorems for integrated processes, it appears likely that the results can be extended to conditionally heteroskedastic processes as well. For the stationary cases this follows directly from the arguments in [18], while for integrated processes results (using different assumptions on the innovations) given for example in [25] can be used. The conditions of [25] hold for example in a large number of GARCH type processes, see [26]. The combination of the different sets of assumptions on the innovations is not straightforward, however, and hence would further complicate the proofs. We refrain from including them.

It is worth pointing out that due to the block diagonal structure of $A_{\circ }$ the result $\left(\overset{˘}{C}-C_{\circ }\right)D_{x}^{-1}=O_{P}\left({\left(\log T\right)}^{a}\right)$ implies consistency of the blocks ${\overset{˘}{C}}_{j}$ corresponding to unit root $z_{j}$ (or the corresponding complex pair) of order almost $T^{-1}$. Using the complex valued canonical form this implies consistent estimation of $C_{\circ ,j,C}$ by the corresponding ${\overset{˘}{C}}_{j,C}$. In the canonical form this matrix determines the cointegrating relations (both the static as well as the dynamic ones, for details see [15]) as the unitary complement to this matrix. It thus follows that CVA delivers estimators for the cointegrating relations at the various unit roots that are (super-)consistent. In fact, the proof can be extended to show convergence in distribution of $\left(\overset{˘}{C}-C_{\circ }\right)D_{x}^{-1}$. This distribution could be used in order to derive tests for cointegrating relations. However, preliminary simulations indicate that these estimates and hence the corresponding tests are not optimal and can be improved upon by quasi maximum likelihood estimation in the VARMA setting initialized by the CVA estimates. Therefore we refrain from presenting these results.

Note that the assumptions impose the restriction $\rho_{0}>0$ excluding VAR systems. This is done solely for stating a uniform lower bound on the increase of *p* as a function of *T*. This bound is related to the lag length selection achieved using BIC, see [27]. In the VAR case the lag length estimator using BIC will converge to the true order and thus remain finite. All results hold true if in the VAR case a fixed (that is independent of the sample size) p≥n is used.

## 5. Inference Based on the Subspace Estimators

Beside consistency of the impulse response sequence also the specification of the integers $c_{0},...,c_{S/2}$ is of interest. First, following [20] this information can be obtained by detecting the unity singular values in the RRR step of the procedure. Second, from the system representation (2) it is clear that the location of the unit roots is determined by the eigenvalues of $A_{\circ }$ on the unit circle: The integers $c_{j}$ denote the number of eigenvalues at the corresponding locations on the unit circle (provided the eigenvalues are simple). Due to perturbation theory (see for example Lemma A2) we know that the eigenvalues of $\hat{A}$ will converge (for T→∞) to the eigenvalues of $A_{\circ }$ and the distribution of the mean of all eigenvalues of $\hat{A}$ converging to an eigenvalue of $A_{\circ }$ can be derived based on the distribution of the estimation error $\hat{A}-A_{\circ }$. This can be used to derive tests on the number of eigenvalues at a particular location on the unit circle. Third, if n≤s the state process is a VAR(1) process and hence in some cases allows for inference on the number of cointegrating relations and thus also on the integers $c_{j}$ as outlined in [4]. Tests based on these three arguments will be discussed below.

**Theorem** **2.** *Under the assumptions of Theorem 1 the test statistic $T\sum_{i=1}^{c}\left(1-{\hat{\sigma }}_{i}^{2}\right)$ converges in distribution to the random variable*

$$Z=tr\left[E\left({\tilde{\varepsilon }}_{t,⊥}{\tilde{\varepsilon }}_{t,⊥}^{\prime }\right){\left(\int_{0}^{1}W\left(r\right)W{\left(r\right)}^{\prime }\right)}^{-1}\right]$$

*where ${\tilde{\varepsilon }}_{t,⊥}={\tilde{\varepsilon }}_{t,1}-E{\tilde{\varepsilon }}_{t,1}{\tilde{\varepsilon }}_{t,•}^{\prime }{\left(E{\tilde{\varepsilon }}_{t,•}{\tilde{\varepsilon }}_{t,•}^{\prime }\right)}^{-1}{\tilde{\varepsilon }}_{t,•}$ (for definition of ${\tilde{\varepsilon }}_{t,1}$ and ${\tilde{\varepsilon }}_{t,•}$ see the proof in Appendix C.2) and where W(r) denotes a c-dimensional Brownian motion with variance*

$$\sum_{i=0}^{S-1}A_{u}^{i}K_{u}\Omega K_{u}^{\prime }{\left(A_{u}^{i}\right)}^{\prime }$$

*with $A_{u}$ denoting the c×c heading submatrix of A and $K_{u}$ denoting the submatrix of K composed of the first c rows such that $\left(A_{u},C_{u},K_{u}\right)$ denotes the unstable subsystem.*

The theorem is proved in Appendix C.2, where also the many nuisance parameters of the limiting random variable are explained and defined. The proof also corrects an error in Theorem 4 of [20], where the wrong distribution is given since the second order terms were neglected.

As the distribution is not pivotal and in particular contains information that is unknown when performing the RRR step, it is not of much interest for direct application. Nevertheless the order of convergence allows for the derivation of simple consistent estimators of the number of common trends: Let ${\hat{c}}_{T}$ denote the number of singular values calculated in the RRR that exceed $\sqrt{1-h(T)/T}$ for arbitrary h(T)→∞,h(T)<T,h(T)/T→0, for T→∞. Then it is a direct consequence of Theorem 2 in combination with ${\hat{\sigma }}_{j}\to \sigma_{j}<1,j>c$, that ${\hat{c}}_{T}\to c$ in probability, implying consistent estimation of *c*. Based on these results also estimators for *c* could be derived, for example along the lines of [28]. However, as [29] shows, such estimators have not performed well in simulations and thus are not considered subsequently.

The singular values do not provide information on the location of the unit roots. This additional information is contained in the eigenvalues of the matrix $A_{\circ }$:

**Theorem** **3.** *Under the assumptions of Theorem 1 let ${\hat{\lambda }}_{i}\left(m\right),i=1,...,c_{m}$ denote the $c_{m}$ eigenvalues of $\hat{A}$ closest to the unit root $z_{m},\left|z_{m}\right|=1$. Then defining ${\hat{\mu }}_{m}=\sum_{i=1}^{c_{m}}\left({\hat{\lambda }}_{i}\left(m\right)-z_{m}\right)$ we obtain*

$$T{\hat{\mu }}_{m}\overset{d}{\to }tr\left[{\left(\int B(r)B(r)dr\right)}^{-1}\int B\left(r\right)dB{\left(r\right)}^{\prime }\right]$$

*where B(r) denotes a $c_{m}$-dimensional Brownian motion with zero expectation and variance $I_{c_{m}}$ for $z_{m}=\pm 1$ and a complex Brownian motion with expectation zero and variance equal to the identity matrix else.*

*Further if $\tilde{A}:=\langle x_{t+1},x_{t}\rangle {\langle x_{t},x_{t}\rangle }^{-1}$ using the true state $x_{t}$ and ${\tilde{\mu }}_{m}=\sum_{i=1}^{c_{m}}\left({\tilde{\lambda }}_{i}\left(m\right)-z_{m}\right)$ where ${\tilde{\lambda }}_{i}\left(m\right),i=1,...,c_{m}$ denote the $c_{m}$ eigenvalues of $\tilde{A}$ closest to $z_{m}$, then $T\left({\hat{\mu }}_{m}-{\tilde{\mu }}_{m}\right)=o_{P}\left(1\right).$*

Therefore the estimated eigenvalues can be used in order to obtain a test on the number of common trends at a particular frequency for each frequency separately. The test distribution is obtained as the limit to

$$Ttr[\langle K_{\circ ,m,C}\varepsilon_{t},x_{t,m,C}\rangle {\langle x_{t,m,C},x_{t,m,C}\rangle }^{-1}]$$

where $x_{t,m,C}=\bar{z_{m}}x_{t-1,m,C}+K_{\circ ,m,C}\varepsilon_{t-1},x_{1,m,C}=0$. The distribution thus does not depend on the presence of other unit roots or stationary components of the state. Furthermore it can be seen that it is independent of the noise variance or the matrix $K_{\circ ,m,C}$. Hence critical values are easily obtained from simulations. Also note that the limiting distribution is identical for all complex unit roots.

Therefore, for each seasonal unit root location $z_{m}$ we can order the eigenvalues of the estimated matrix $\hat{A}$ with increasing distance to $z_{m}$. Then starting from the assumption of $H_{0}:c_{m}=\bar{c}$ (for a reasonable $\bar{c}$ obtained, e.g., from a plot of the eigenvalues) one can perform the test with statistic $T{\hat{\mu }}_{m}$. If the test rejects, then the hypothesis $H_{0}:c_{m}=\bar{c}-1$ is tested, until the hypothesis is not rejected anymore, or $H_{0}:c_{m}=1$ is reached. This is then the last test. If $H_{0}$ is rejected again, no unit root is found at this location. Otherwise we do not have evidence against $c_{m}=1$. In any case, the system needs to be estimated only once and the calculation of the test statistics is easy even for all seasonal unit roots jointly.

The third option for obtaining tests is to use the tests derived in [4] based on the JS framework for VARs. In the case n≤s the state process $x_{t+1}=Ax_{t}+K\varepsilon_{t}$ is a seasonally integrated VAR(1) process (for n>s the noise variance is singular). The corresponding VECM representation equals

$$p\left(L\right)x_{t}=\sum_{m=1}^{S}\left(I_{n}-Az_{m}\right)X_{t-1}^{\left(m\right)}+K\varepsilon_{t-1}=\sum_{m=1}^{S}\alpha_{m}\beta_{m}^{\prime }X_{t-1}^{\left(m\right)}+K\varepsilon_{t-1}$$

where $z_{m}=\exp\left(\frac{2\pi m}{S}i\right),m=1,...,S$ and

$$\begin{array}{ccc} p\left(L\right)=1-L^{S} & , & p_{t}=p\left(L\right)x_{t}=x_{t}-x_{t-S},\\ p_{m}\left(L\right)=\frac{p(L)}{1-\bar{z_{m}}L} & , & X_{t}^{\left(m\right)}=-\frac{p_{m}\left(L\right)}{p_{m}\left(z_{m}\right)z_{m}}x_{t}. \end{array}$$

Note that in this VAR(1) setting no additional stationary regressors of the form $p\left(L\right)x_{t-j}$ occur. Also no seasonal dummies are needed but could be added to the equation. In this setting [4] suggests to use the eigenvalues ${\hat{\lambda }}_{i}$ (ordered with increasing modulus) of the matrix (the superscript ${\left(.\right)}^{\pi }$ denotes the residuals with respect to the remaining regressors $X_{t-1}^{\left(j\right)},j\neq m$)

$$\langle X_{t-1}^{(m),\pi },p_{t}^{\pi }\rangle {\langle p_{t}^{\pi },p_{t}^{\pi }\rangle }^{-1}\langle p_{t}^{\pi },X_{t-1}^{(m),\pi }\rangle {\langle X_{t-1}^{(m),\pi },X_{t-1}^{(m),\pi }\rangle }^{-1}$$

as the basis for a test statistic

$${\tilde{C}}_{m}:=-\delta_{m}\sum_{i=1}^{c_{m}}\log\left(1-{\hat{\lambda }}_{i}\right).$$

where $\delta_{m}=2$ for complex unit roots and $\delta_{m}=1$ for real unit roots. In the I(1) case this leads to the familiar Johansen trace test, for seasonal unit roots a different asymptotic distribution is obtained.

**Theorem** **4.** *Under the assumptions of Theorem 1 let ${\hat{C}}_{m}$ be calculated based on the estimated state and let ${\tilde{C}}_{m}$ denote the same statistic based on the true state. Then for n≤s it holds that ${\hat{C}}_{m}-{\tilde{C}}_{m}=o_{P}\left(T^{-1}\right)$ and*

$$T{\hat{C}}_{m}\overset{d}{\to }tr\left[\int dB\left(r\right)B{\left(r\right)}^{\prime }{\left(\int B(r)B(r)dr\right)}^{-1}\int B\left(r\right)dB{\left(r\right)}^{\prime }\right]$$

*where B(r) is a real Brownian motion for $z_{m}=\pm 1$ or a complex Brownian motion else.*

Thus again under the null hypothesis the test statistic based on the estimated state and the one based on the true state reject jointly asymptotically with probability one. Therefore for n≤s the tests of JS can be used to obtain information on the number of common cycles, ignoring the fact that the estimated state is used in place of the true state process.

After presenting three disjoint ideas for providing information on the number and location of unit roots, the question arises, which one to use in practice. In the following a number of ideas are given in this respect.

The criterion based on the singular values given in Theorem 2 is of limited information as it only provides the overall number of unit roots. Since the limiting distribution is not pivotal it cannot be used for tests and the choice of the cutoff value h(T) is somewhat arbitrary. Nevertheless, using a relatively large value one obtains a useful upper bound on *c* which can be included in the typical sequential procedures for tests for $c_{j}$.

Using the results of Theorem 4 has the advantage of using a framework that is well known to many researchers. It is remarkable that in terms of the asymptotic distributions there is no difference involved in using the estimated state in place of the true state. The assumption n≤s, however, is somewhat restrictive except in situations with a large *s*.

Finally the results of Theorem 3 provide simple to use tests for all unit roots, independently of the specification of the model for the remaining unit roots. Again it is remarkable that, under the null, inference is identical for known and for estimated state.

Since our estimators are not quasi maximum likelihood estimators the question of a comparison with the usual likelihood ratio tests arises. For VAR models simulation exercises documented in Section 7 below demonstrate that there are situations where the proposed tests outperform tests in the VAR framework. Comparisons with tests in the state space framework (or equivalently in the VARMA framework) are complicated by the fact that no results are currently available in the literature of this framework. One difference, however, is given by the fact that quasi likelihood ratio tests in the VARMA setting require a full specification of the $c_{j}$ values for all unit roots. This introduces interdependencies such that the tests for one unit root depend on the specification of the cointegrating rank at the other roots. The interdependencies can be broken by performing tests based on alternative specifications for each unit root. The test based on Theorem 3 does not require this but can be performed based on the same estimate $\hat{A}$. This is seen as an advantage.

The question of the comparison of the empirical size in finite samples as well as power to local alternatives between the CVA based tests and tests based on quasi-likelihood ratios is left as a research question.

## 6. Deterministic Terms

Up to now it has been assumed that no deterministic terms appear in the model contrary to common practice. In the VAR framework dealing with trends is complicated by the usage of the VECM representation, see e.g., [30]. In the state space framework used in this paper, however, deterministic terms are easily incorporated.

**Theorem** **5.** *Let the process ${\left(y_{t}\right)}_{t\in Z}$ be generated according to Assumption 1 and assume that the process ${\left({\tilde{y}}_{t}\right)}_{t\in Z}$ is observed where ${\tilde{y}}_{t}=y_{t}+\Phi d_{t}$ with*

$$d_{t}={\left[\begin{array}{ccccc} 1, & \cos(\frac{2\pi }{S}t), & \sin(\frac{2\pi }{S}t), & \cdots & {\left(-1\right)}^{t} \end{array}\right]}^{\prime }\in R^{S}$$

*and $\Phi \in R^{s\times S}$.*

*Then if the* CVA *estimation is applied to*

$${\tilde{y}}_{t}^{\pi }:=y_{t}-\left(\sum_{t=1}^{T}y_{t}d_{t}^{\prime }\right){\left(\sum_{t=1}^{T}d_{t}d_{t}^{\prime }\right)}^{-1}d_{t},\;t=1,...,T,$$

*the results of Theorem 1 hold, i.e., the system is estimated consistently and the orders of convergence for the transformed system $\left(\overset{˘}{A},\overset{˘}{C},\overset{˘}{K}\right)$ hold true.*

*Furthermore the convergence in distribution results in Theorems 2–4 hold true where in the limits the Brownian motions B(r) occurring in the distributions must be replaced by their demeaned versions $B\left(r\right)-\int_{0}^{1}B\left(s\right)ds$.*

In this sense the results are robust to some operations typically termed preprocessing of data such as demeaning and deseasonalizing using seasonal dummies. More general preprocessing steps such as detrending or the extraction of more general deterministic terms analogous to [30] can be investigated along the same lines.

## 7. Simulations

The estimation of the seasonal cointegration ranks and spaces is usually carried out via quasi maximum likelihood methods that originated from the VAR model class. Typical estimators in this setting are those of [2,4,5,31]. In the first two experiments we focus on the estimation of the cointegrating spaces and the specification of the cointegration ranks in the classical situation of quarterly data and show that there are certain situations in which CVA estimators and the test in Theorem 3 possess finite sample properties superior to those of the methods above. In a third experiment the test performance is evaluated for a daily sampling rate. Moreover, the prediction accuracy of CVA is investigated as well as its robustness to innovations exhibiting behaviors often encountered at such higher sampling rates. All simulations are carried out using 1000 replications.

To investigate the practical usefulness of the proposed procedures we generate quarterly data using two VAR dgps of dimension s=2 first and then two more general VARMA dgps with s=8. Each pair contains dgps with different state space unit root structures

$$\left\{\left(0,\left(1\right)\right),\left(\pi /2,\left(c_{\pi /2}\right)\right),\left(\pi ,\left(1\right)\right)\right\},\;c_{\pi /2}=1,2.$$

From all four dgps samples of size T∈{50,100,200,500} are generated with initial values set to zero. Although none of the dgps contains deterministics, the data is adjusted for a constant and quarterly seasonal dummies as in [5]. For reasons of comparability, the adjustment for deterministic terms is done before estimation.

In the third experiment we generate daily data with dimension s=4 from a state space system including unit roots corresponding to weekly frequencies (that is a period length of seven days). In the simulations we use several years of data (excluding new year’s day to account for 52 weeks of seven days each). The first 200 observations are discarded to include the effects of different starting values. In this example the focus lies on a comparison of the prediction accuracy. Furthermore we investigate the robustness of the test procedures to conditional heteroskedasticity of the GARCH type as well as to non-normality of the innovations.

To assess the performance of specifying the cointegrating rank at unit root *z* using CVA, the following test statistic is constructed from the results in Theorem 3

(6) $$\Lambda \left(c\right)=T|\left(\frac{1}{c}\sum_{i=1}^{c}{\hat{\lambda }}_{i}\right)-z|\;.$$

Here ${\hat{\lambda }}_{1},\ldots ,{\hat{\lambda }}_{n}$ are the eigenvalues of $\hat{A}$ ordered increasingly according to the distance from *z*. Note that a similar test in [20] only uses the *c*-th largest eigenvalue, whereas here the average over the nearest *c* eigenvalues is taken. Critical values have been obtained by simulation using large sample sizes (sample size 2000 (JS) and 5000 (CVA), 10,000 replications).

In our first two experiments usage of Λ(c) is compared with variants of the likelihood ratio test from [2] (JS), [4] ($Q_{1}$), and [5] ($Q_{2}$, $Q_{3}$). $Q_{1}$ is Cubadda’s trace test for complex-valued data, $Q_{2}$ takes the information at frequency π/2 into account when the analysis is carried out at frequency 3π/2, and $Q_{3}$ iterates between π/2 and 3π/2 in the alternating reduced rank regression (ARR) of [5]. For the procedure of [2] the likelihood maximization at frequency π/2 is carried out using numerical optimization (BFGS) with initial values obtained from an unrestricted regression.

All tests are evaluated by comparing the percentages of correctly detected common trends, or *hit rates*, with 0.95, the hit rate to be expected from a nominal significance level of 0.05. The testing procedure employed for all tests is the same: at each of the frequencies it is started from a null hypothesis of *s* unit roots against less than *s* unit roots. In case of rejection, s−1 unit roots are tested versus less than s−1 and so on, until there are zero unit roots under the alternative.

For the first two experiments the estimation performance of CVA for the simultaneous estimation of the seasonal cointegrating spaces is compared with the maximum likelihood estimates of [2,4,31] (cRRR), and also with an iterative procedure (Generalized ARR or GARR) of [5]. The comparison is carried out by means of the gap metric, measuring the distance between the true and the estimated cointegrating space as in [32]. The smaller the mean gap over all replications, the better is the estimation performance. Throughout a difference between two mean gaps or two hit rates is considered statistically significant if it is larger than twice the Monte Carlo standard error.

For all procedures used in this section, an AR lag length has to be chosen first. For CVA this can be done using the AIC as in ([33], Section 5), as is done in the third experiment.

In the first two experiments where sample sizes are rather small, we estimate the lag length via minimization of the corrected AIC (AICc) ([34], p. 432), ${\hat{k}}_{AICc}$, benefitting the simulation results. For larger sample sizes the two criteria lead to the same choices. Due to the quarterly data we work with, the lag length is then chosen to be $\hat{k}=\max\left\{{\hat{k}}_{AICc},4\right\}$.

Other information criteria could be chosen here. An anonymous referee also suggested the application of the Modified Akaike Information Criterion (MAIC) of [35], proposed there for the I(1)-case. In an attempt to apply it to the seasonally integrated case considered here, it performed considerably worse than the AICc. Thus we refrain from using the MAIC in the following and also omit the results of that attempt. They can be obtained from the authors upon request.

For CVA the truncation indices *f* and *p* are chosen as $\hat{f}=\hat{p}=2\hat{k}$ ([33], Section 5). The system order *n* is estimated by minimizing ([33], Section 5)

(7) $$SVC\left(n\right)={\hat{\sigma }}_{n+1}^{2}+2ns\frac{\log T}{T}\;.$$

Here ${\hat{\sigma }}_{i}$ denotes the *i*-th largest singular value from the singular value decomposition of ${\hat{\Xi }}_{f}^{+}{\hat{\beta }}_{1}{\hat{\Xi }}_{p}^{-}$ (Step 2 of CVA). Note that selecting the number of states by SVC is made less influential insofar as $\hat{n}=\max\left\{c_{0}+2c_{\pi /2}+c_{\pi },{\hat{n}}_{SVC}\right\}$, where ${\hat{n}}_{SVC}$ denotes the SVC estimated system order.

In Section 7.1 we start with the two VAR dgps and find that the likelihood-based procedures are mostly superior. Continuing with the VARMA dgps in Section 7.2, CVA performs better and is superior for the smaller sample sizes in terms of size and gap and better for all sample sizes in terms of power. Section 7.3 evaluates the performance of the tests for unit roots for larger sample sizes together with the prediction performance in this setting. We find that the tests are robust to the distribution of the innovations as well as to conditional heteroskedasticity of the GARCH type. Furthermore the empirical size of the tests lies close to the size already for moderate sample sizes, where the tests also show almost perfect power properties.

### 7.1. VAR Processes

The VAR dgps considered in this paper are given by,

(8) $$X_{t}=\Pi_{1}X_{t-1}+\Pi_{2}X_{t-2}+\Pi_{3}X_{t-3}+\Pi_{4}X_{t-4}+\varepsilon_{t},\;\varepsilon_{t}∼N\left(\left[\begin{array}{c} 0\\ 0 \end{array}\right],\left[\begin{array}{cc} 1 & 0.5\\ 0.5 & 1 \end{array}\right]\right)$$

where ${\left(\varepsilon_{t}\right)}_{t\in Z}$ is white noise and the coefficient matrices are

$$\begin{array}{ccc} \Pi_{1} & = & \left[\begin{array}{cc} \gamma & 0\\ 0 & 0 \end{array}\right],\;\Pi_{2}=\left[\begin{array}{cc} -0.4 & 0.4-\gamma \\ 0 & 0 \end{array}\right],\\ \Pi_{3} & = & \left[\begin{array}{cc} -\gamma & 0\\ 0 & 0 \end{array}\right],\;\Pi_{4}=\left[\begin{array}{cc} 0.6-(\gamma /10) & 0.4+\gamma \\ 0 & 1 \end{array}\right]. \end{array}$$

This dgp is adopted from [5] with a slight adjustment to $\Pi_{4}$. The corresponding VECM representation in the notation of [5] equals

$$\begin{array}{ccc} X_{0,t} & = & \left[\begin{array}{c} -0.2\\ 0 \end{array}\right]\left[\begin{array}{cc} 1+\gamma /8 & -1 \end{array}\right]X_{1,t-1}+\left[\begin{array}{c} 0.2\\ 0 \end{array}\right]\left[\begin{array}{cc} 1+\gamma /8 & -1 \end{array}\right]X_{2,t-1}+\\ & & \left[\begin{array}{c} \gamma \\ 0 \end{array}\right]\left[\begin{array}{cc} 1+0.05L & -L \end{array}\right]X_{3,t-1}+\varepsilon_{t}. \end{array}$$

As can be seen from Table 1, the dgps possess unit roots at frequencies 0, π, and π/2, where $c_{\pi /2}=2\left[1\right]$ for γ=0[0.2], respectively. Note that in all cases the order of integration equals 1, while the number of common cycles at π/2 is varied.

Table 2 exhibits the hit rates from the application of the different test statistics. At frequencies 0 and π, Λ is compared with the trace test of Johansen (J; based on [31] for unit roots z=−1), whereas at π/2 it is competing with JS, $Q_{1},Q_{2}$, and $Q_{3}$. All competitors are likelihood-based tests which is the term we are referring to when we compare Λ to them as a whole.

The results for 0 and π are very similar for both dgps in that Λ scores more hits than the likelihood-based tests when the sample size is small, T∈{50,100}. Convergence of its finite sample distribution is slower than for the other test statistics, however, as J is closer to 0.95 from T=200 on. For T=500 the distribution of Λ only seems to have converged to its asymptotic distribution when $c_{\pi /2}=2$ at frequency 0, whereas convergence of the likelihood-based tests has occurred in all cases.

At π/2 the likelihood ratio test of JS strictly dominates all implementations of [5] for all sample sizes and both dgps. It strictly dominates the CVA-based test procedure as well, except for one case, it seems: when $c_{\pi /2}=1$ and T=50Λ scores slightly, but significantly, more hits than the likelihood ratio test of JS. Surprisingly, Λ is drastically worse when T=100 with only 8.7%, only to be up at 85% for T=200.

The behavior of Λ is explained by $z_{5}$ and $z_{6}$ being close to ±i when $c_{\pi /2}=1$, cf. Table 1. For future reference we will call the corresponding roots *false unit roots*.

**Figure 1:**
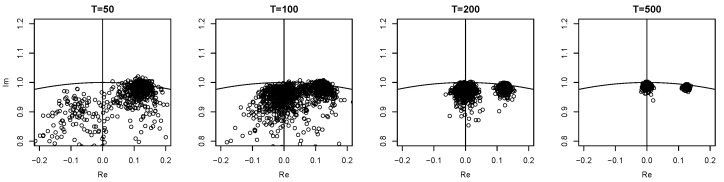
Eigenvalues around z=i of 1000 replications when γ=0.2 ($c_{\pi /2}=1$).

For T=50 the estimates of eigenvalues corresponding to actual unit roots are rather not very close to ±i in contrast to the false unit roots. Thus the latter are mistaken for actual unit roots (cf. the first panel in Figure 1), leading to a hit rate of 81.1%, one that is even larger than the rates at 0 and π. As the sample size increases, the eigenvalue estimates of the true unit roots become more and more accurate, visible from the second and third panel in Figure 1. Accordingly they can be detected correctly more often. Unfortunately however, for T=100 the false unit roots remain to be detected such that often two instead of just one unit root are found by Λ, resulting in a hit rate of only 8.7%. For T∈{200,500}Λ is able to distinguish the false unit roots from the true ones and the detection rate is getting closer to the asymptotic rate, 85.5% and 92.7%, respectively.

When the VAR dgp without false unit roots and $c_{\pi /2}=2$ is considered, it is visible that the hit rates of Λ at π/2 are monotonously increasing in the sample size again. The rates are smaller than those of the likelihood-based tests, however, and also clearly worse than those of Λ at 0 and π, cf. Table 2 again.

Taken together, at frequencies 0 and π which correspond to real-valued unit roots, the use of Λ was advantageous for T=50. It also scored more hits for T=100 and $c_{\pi /2}=1$. For higher sample sizes the likelihood-based tests clearly dominate Λ at these two frequencies. At π/2 this superiority of the likelihood-based tests for all sample sizes and both dgps continues. The example also points to a general weakness: if the sample size is low and *false unit roots* are present, it can be difficult for Λ to distinguish them from actual unit roots.

### 7.2. VARMA Processes

The second setup consists of VARMA data generated by a state space system $(A_{r},C_{r},K_{r}),$ r=1,2, as in (1), where the matrices $A_{1}$ and $A_{2}$ are constructed as in (2) and are taken to be

(9) $$\begin{array}{c} A_{1}=\left[\begin{array}{cccc} 1 & 0 & 0 & 0\\ 0 & -1 & 0 & 0\\ 0 & 0 & 0 & 1\\ 0 & 0 & -1 & 0 \end{array}\right],\;A_{2}=\left[\begin{array}{cccccc} 1 & 0 & 0 & 0 & 0 & 0\\ 0 & -1 & 0 & 0 & 0 & 0\\ 0 & 0 & 0 & 1 & 0 & 0\\ 0 & 0 & -1 & 0 & 0 & 0\\ 0 & 0 & 0 & 0 & 0 & 1\\ 0 & 0 & 0 & 0 & -1 & 0 \end{array}\right]\;. \end{array}$$

These two choices yield the same state space unit root structures as those of the two VAR dgps with $c_{\pi /2}=1$ and $c_{\pi /2}=2$ for $A_{1}$ and $A_{2}$, respectively. The other two system matrices $K_{r}\in R^{(2+2r)\times s}$ and $C_{r}\in R^{s\times (2+2r)}$ with s=8 are drawn randomly from a standard normal distribution in each replication and ${\left(\varepsilon_{t}\right)}_{t\in Z}$ is multivariate normal white noise with an identity covariance matrix.

Note that these systems are within the VARMA model class such that the dgp is contained in the VAR setting only by increasing the lag length as a function of the sample size. While superiority of the CVA approach in such a setting might be expected, this is far from obvious. Moreover, using a long VAR approximation is the industry norm in such situations.

From the hit rates in Table 3 it can be seen that the combination of large *s*, small *T*, and a minimal lag length of four render the likelihood-based tests useless at all frequencies with hit rates below ten percent for T=50. Λ in contrast does not suffer from this problem and is already close to 95% for this sample size. Only when T=200 do the likelihood-based tests appear to work, exhibiting hit rates close to 95%.

**Figure 2:**
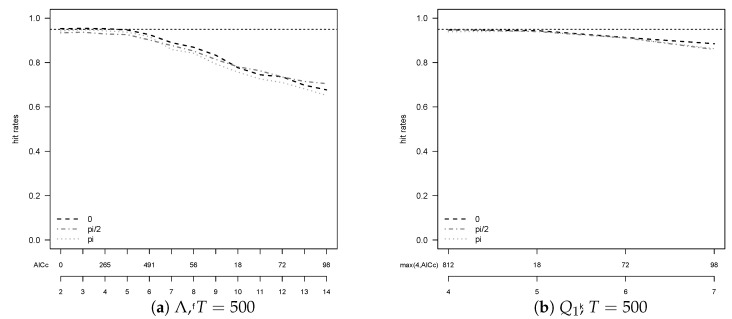
Relationship between hit rates and chosen values of *f* and *k*, illustration for the VARMA dgp using *A_2_*. The lower x-axes show *f* or *k*, above are the choice frequencies of the selection criteria.

For all tests alike, however, it is striking that hit rates move away from 95% when T=500. This behavior is most pronounced for Λ, e.g., from T=200 to T=500 its hit rate drops from 93.1% to 82.4% at 0 when $A_{2}$ is used. This phenomenon is a consequence of the fact that *f* and *k* in the algorithm are chosen data dependent. An inspection of how the hit rates depend on *f* and *k* and a comparison with the actually selected $\hat{f},\hat{k}$ reveals that for T=500 too large values of *f* and *k* are chosen too often and leave room for improvement in the hit rates, cf. Figure 2. The figure stresses an important point: The performance of the unit root tests is heavily influenced by the selected lag lengths for all procedures. We tested a number of different information criteria in this respect. AICc turned out to be the best criterion overall, but not uniformly. Figure 2 indicates advantages for this example of BIC over AIC as it on average selects smaller lag lengths, associated here with higher hit rates.

**Figure 3:**
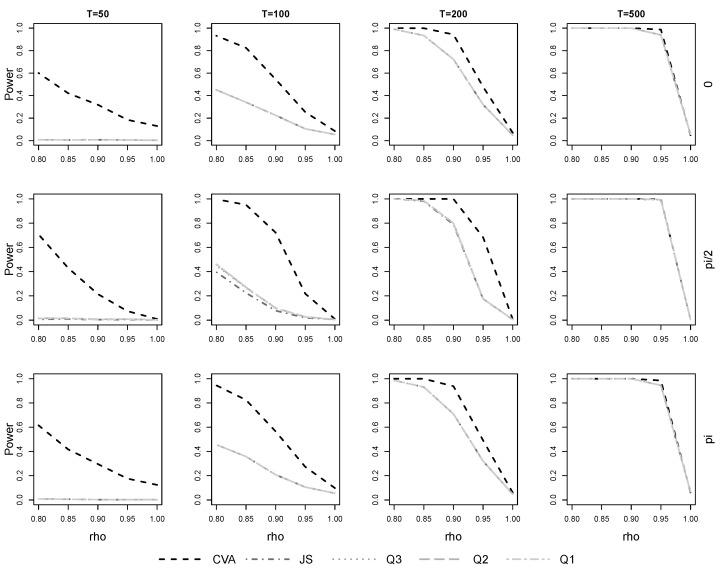
Empirical power of the different test procedures (VARMA dgp with $A_{2}$). Twice the Monte Carlo standard error is 0.005.

To study the power of the different procedures, the transition dynamics $A_{r}$ in (9) are multiplied by ρ∈{0.8,0.85,0.9,0.95} so that the systems do not contain unit roots at any of the frequencies. Here empirical power is defined as the frequency of choosing zero common trends. This is why for ρ=1, when there are in fact common trends present in our specifications, the empirical power values plotted in Figure 3 are not equal to the actual size we could define as one minus the hit rate: our measure of empirical power in this situation only counts the false test conclusion of zero common trends, but there are of course multiple ways the testing procedure could conclude falsely.

As expected, rejection of the null hypothesis is easiest when ρ is small and is very difficult when it is close to 1, cf. Figure 3 for the case of $A_{2}$.

Further, there are almost no differences among the likelihood-based tests over all combinations of sample size and frequency, only for T=100 is JS significantly worse than the $Q_{i},i=1,2,3$ at π/2. It is also clearly visible at all frequencies that the likelihood-based tests possess no or only very limited power when T=50 and T=100, respectively. Λ, in contrast, is clearly more powerful in these cases. As the sample size increases to T=200, the power of each test improves, still Λ remains the most powerful option. Only for T=500 have the differences almost vanished with small, but significant, advantages for Λ at 0 and π.

The results are the same when $A_{1}$ is used and $c_{\pi /2}=1$ and all of the differences described here are statistically significant.

Next the estimation performance of CVA is evaluated by calculation of the gaps between the true and the estimated cointegrating spaces. At all frequencies these gaps are compared with the GARR procedure of [5] which cycles through frequencies. At π/2 CVA and GARR are also compared with our implementation of JS and cRRR of [4], whereas it is also compared with the usual Johansen procedure at 0 and π. All estimates are conditional on the true state space unit root structure in the sense that the minimal number of states used is larger or equal to the number of unit roots over all frequencies. Other than imposing a minimum state dimension, the estimation of the order using SVC is not influenced. The likelihood-based procedures, on the other hand, take the unit root structure as given, i.e., do not perform CI rank testing for this estimation exercise.

From the results in Table 4 it can be noted first that the likelihood-based procedures show mostly equal mean gaps. Only for π/2 and T=50 and both dgps does JS possess significantly larger gaps than cRRR and GARR and other differences are not statistically significant. Thus it does not matter in our example whether the iterative procedure is used or not.

Second, CVA is again superior for T=50 where it exhibits mean gaps that are significantly smaller than those of the other estimators at all frequencies. This advantage is turned around for higher sample sizes, though: mean gaps are smaller for the likelihood-based procedures when T∈{100,200,500} and $A_{2}$ is used, if only slightly. When $A_{1}$ is used instead, mean gaps do not differ significantly from each other at π/2 when T>50 and at 0,π when T=100 and those of CVA are only very modestly worse when T∈{200,500} at 0,π.

Thus, when it comes to estimating the cointegrating spaces, CVA is superior for T=50 and equally good or only slightly worse than the likelihood-based procedures for higher sample sizes. For the systems analyzed, decreasing $c_{\pi /2}$ leads to gaps that are smaller for all methods and these improvements are slightly larger for CVA than for the other estimators.

### 7.3. Robustness of Unit Root Tests for Daily Data

In this last simulation example we examine the robustness of the proposed procedures with regard to test performance and prediction accuracy with respect to the innovation distribution and the existence of conditional heteroskedasticity of the GARCH-type, as these features are often observed in data of higher frequency, for example in financial applications. While our asymptotic results do not depend on the distribution of the innovations (subject to the assumptions), the assumptions do not include GARCH effects. Nevertheless, the theory in [25,26] suggests that the tests might be robust also in this respect.

We generate a state space system of order n=8 using the matrix $A={\left[A_{i,j}\right]}_{i,j=1,...,8}$ where $A_{i,i+1}=1,i=1,...,6,A_{7,1}=1,A_{8,8}=0.8$ and $A_{i,j}=0$ else. This implies that the eigenvalues of this matrix are $\lambda_{j}=\exp\left(2\pi ij/7\right),j=1,...,7,\lambda_{8}=0.8$. Therefore the corresponding process has state space unit root structure

((0,(1)),(2π/7,(1)),(4π/7,(1)),(6π/7,(1))).

The entries of the matrices *C* and *K* are chosen as independent standard normally distributed random variables as before.

A process ${\left(y_{t}\right)}_{t=1,...,T}$ is generated from filtering an independent identically distributed innovation process ${\left(\varepsilon_{t}\right)}_{t=-199,...,T+1}$ through the system (A,C,K). The first 200 observations are discarded, the last are used for validation purposes. A total of 1000 replications are generated where in each replication a different system is chosen.

With the generated data three different estimates are obtained: An autoregressive model (called AR in the following) is estimated with lag length chosen using AIC of maximal lag length equal to $\left\lfloor \sqrt{T}\right\rfloor$. Second, an autoregressive model with large lag length (called ARlong) is estimated. This estimate is used to hint at the behavior of an autoregression using the lag length equal to a full year. This would correspond to estimating a VECM without rank restrictions, when accounting for yearly differences. The third method consists of the CVA estimates, where $f=p=2{\hat{k}}_{AIC}$ is chosen. The order is estimated by minimizing SVC. However, we correct for orders smaller than n=7 which would limit the possibilities of finding all unit roots.

First, we compare the prediction accuracy for the three methods for two different distributions of the innovations: Beside the standard normal distribution also the student t-distribution with v=5 degrees of freedom (scaled to unit variance) is used. This distribution shows considerably heavier tails than the normal distribution but nevertheless is covered by our assumptions.

**Figure 4:**
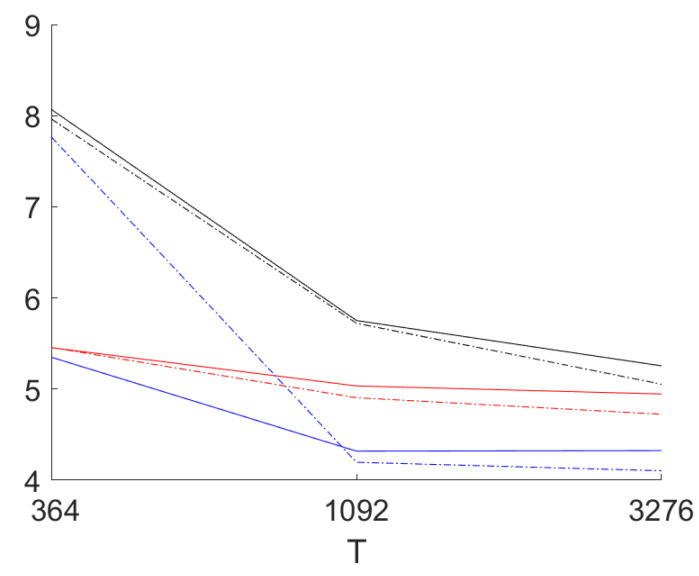
Mean of absolute value of one day ahead prediction error over all four components. CVA (blue), AR (red) and long AR (black). Dash-dot lines refer to the t-distribution.

Figure 4 provides the results for out-of-sample one day ahead mean absolute prediction error (over all coordinates) for the sample sizes T=364 days (one year), T=1092 (3 years) and T=3276 (nine years). The long AR model is estimated with lag lengths of 8 weeks for the smallest sample size, 10 weeks for the medium sample size and 12 weeks for the largest sample size.

In the figure the results for the normally distributed innovations are presented as well as the ones for the t-distributed residuals (scaled to unit variance). It can be seen that for the two larger sample sizes the mean absolute error for the residuals for CVA is smaller in all cases. For the smallest sample size, by contrast, results are mixed. For CVA the results for the heavy tailed distribution in this case are much worse than for the normal distribution. For the larger sample sizes the differences are small. The maximal standard error of the estimated means over 1000 replications for T=1092 and T=3276 amounts to 0.05. This allows the conclusion that CVA performs better for the two larger sample sizes. For T=364 there are no statistically significant differences between the performance of the three methods: CVA seems to suffer more from few very large errors (using the root mean square errors the CVA results are worse for T=364 in comparison; if one uses the 95% percentiles CVA performs best also for the smallest sample size). This results in a standard error over the replications of the mean absolute error for T=364 of 0.18 for normally distributed innovations and 3.4 for t-distributed innovations. The long AR models are clearly worse than the two other approaches. This happens even if we are still far from using a full year as the lag length.

With regard to the unit root tests we investigate results for the tests of the hypotheses $H_{0}:c_{m}=1$ versus $H_{1}:c_{m}=0$ at all frequencies 2πm/364,m=0,...,363. The data generating process features unit roots with $c_{m}=1$ at the seven frequencies 2πk/7,k=0,...,6. Therefore the tests should not reject at these frequencies, but should reject at all others.

Consequently we compare the minimum of the non-rejection rates for the seven unit roots (called empirical size below) as well as the maximum of the non-rejection rates for the non-unit root frequencies $\omega_{j}=2\pi j/364,j\neq 52k,k=0,1,2,...,6$ (called empirical power below).

For the larger sample sizes the empirical size is practically 95% while the empirical power is 100%. For T=364 we obtain an empirical size of 90% for the normal distribution and 91.5% for the t-distribution. The worst empirical power equals 89.3% (normal) and 87.6% (t-distribution). Hence even for one year of data the discrimination properties of the unit root tests are good and we do not observe differences between the normal distribution for the innovations and the heavy tailed t-distribution.

Finally we compare the empirical size and power of the tests for the various unit roots for smaller sample sizes T∈{104,208,312,416,520}. For the experiments we consider univariate GARCH models of the form

$$\varepsilon_{t,i}=h_{t,i}\eta_{t,i},\;h_{t,i}^{2}=1+\alpha \varepsilon_{t-1,i}^{2}+\beta h_{t-1,i}^{2},\;i=1,..,4,$$

where ${\left(\eta_{t,i}\right)}_{t\in Z}$ is independent and identically standard normally distributed. α,β≥0 are reals. It follows that the component processes ${\left(\varepsilon_{t,i}\right)}_{t\in Z}$ show conditional heteroskedasticity, the persistence of which is governed by α+β. Here 0<α+β<1 implies stationarity while α+β=1 implies persistent conditional heteroskedasticity usually termed I-GARCH. We include five different processes for the innovations:

1. norm: α=β=0, no GARCH effects
2. G1: α=0.8,β=0.1
3. IG1: α=0.8,β=0.2
4. IG2: α=0.5,β=0.5
5. IG3: α=0.2,β=0.8

For the five different sample sizes 1000 replications of the estimates using the CVA algorithm are obtained. For each estimate we calculate the test statistic for testing $H_{0}:c_{m}=1$ versus $H_{0}:c_{m}=0$ for m=0,...,363 corresponding to the unit roots $z_{m}=\exp\left(2\pi im/364\right)$. This set of unit roots contains all seven unit roots exp(2πik/7),k=0,...,6.

**Figure 5:**
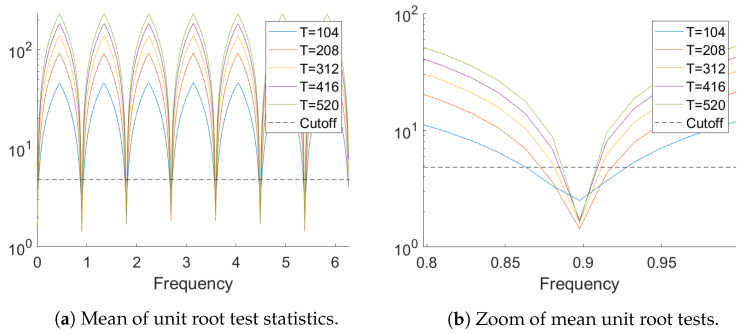
Results of the unit root tests for all seasonal unit roots jointly.

Figure 5 provides the mean over the 1000 replications of the test statistics Λ(1) for $z_{j},j=0,...,363$ and the five sample sizes. It can be seen that the test Λ(1) is able to pinpoint the seven unit roots present in the data generating process fairly accurately even for sample size T=104. The zoom on the region around the unit root frequency 2π/7 shows that the mean value is larger than the cutoff value of the test (the dashed horizontal line) for the adjacent frequency $2\pi \frac{53}{364}$ already for T=312.

Table 5 lists the minimum of the achieved percentages of non-rejections of the test statistic for the seven unit root frequencies as well as the maximum over all non-unit root frequencies. It can be seen that for all GARCH models for T=312 the test rejects unit roots at all non unit root frequencies every time, while the empirical size is close to the nominal 5%. For small sample sizes the tests are slightly undersized while for T=208 a slight oversizing is observed. The two larger sample sizes are omitted as the tests perform perfectly there.

It follows from the examples presented in this subsection that the test is robust also in small samples with respect to heavy tailed distributions of the innovations (subject to the assumptions). Furthermore also a remarkable robustness with respect to GARCH-type conditional heteroskedasticity is observed.

## 8. Application

In this section we apply CVA to the modeling of electricity consumption using a data set from [36]. The dataset contains hourly consumption data (in megawatts) from a number of US regions, scraped from the webpage of PJM Interconnection LLC, a regional transmission organization. The number of regions have changed over time, thus the data set contains many missing values. It also contains data aggregated into regions called east and west, which are not used subsequently.

In order to avoid problems with missing values, we restrict the analysis to four regions, for which data over the same time period is available: American Electric Power (AEP; in the following printed in blue), the Dayton Power and Light Company (DAYTON; black), Dominion Virginia Power (DOM; red) and Duquesne Light Co. (DUQ; green). We use data from 1 May 2005 until 31 July 2018. In this period only 3 data points are missing for the four regions and their imputation is handled by interpolation of the corresponding previous values. One observation in this sample is an obvious outlier which is corrected for analogously.

The data is split into an estimation sample covering observations up to the end of 2016 (102,291 observations on 4263 days) and a validation sample containing data in 2017 and 2018 (13,845 observations on 577 days). Data is equally sampled, but contains two hour segments when switching from winter to summer time or back. Table 6 contains some summary statistics.

**Figure 6:**
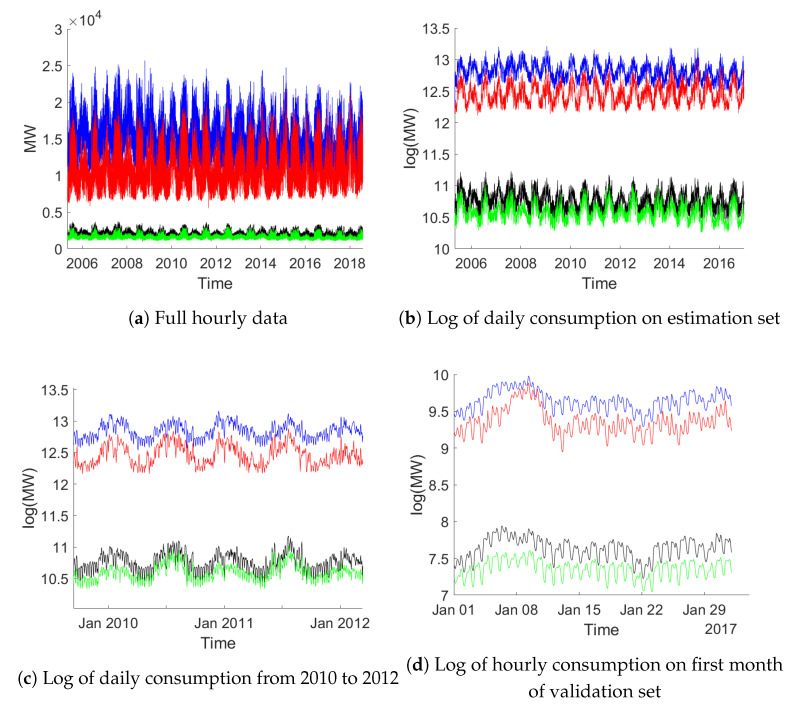
Electricity consumption data.

Figure 6 provides an overview of the data: Panel (a) shows the full data on an hourly basis, while (b) presents aggregation to daily frequency. Panel (c) zooms in on a two year stretch of daily consumption. Panel (d) finally provides hourly data for the first month in the validation data. The figures clearly document strong daily, weekly and yearly patterns. From these figures it appears that these seasonal fluctuations are somewhat regular with changes throughout time. It is hence not clear whether a fixed seasonal pattern is appropriate. Also note that the sampling frequency is on an hourly basis such that a year roughly covers 8760 observations.

In the following we estimate (on the estimation part) and compare (on the validation part) a number of different models, first for the full hourly data set and afterwards for the aggregated daily data. As a benchmark we will use univariate AR models including deterministic seasonal patterns for daily, weekly and yearly variations. Subsequently we estimate models using CVA including different sets of such seasonal patterns.

**Figure 7:**
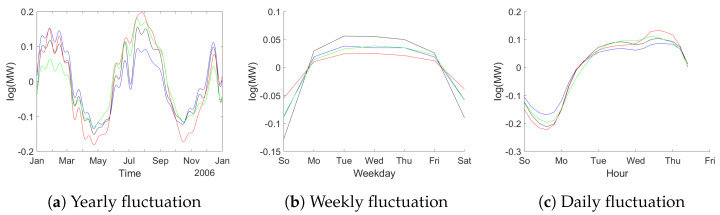
Periodic patterns from dummy variables.

First in the analysis using dummy variables fixed periodic patterns have been estimated. We model the natural logarithm of consumption (to reduce problems due to heteroskedasticity) and include dummies for weekdays, hours and sine and cosine terms corresponding to the first 20 Fourier frequencies with respect to annual periodicity. The corresponding results can be viewed in Figure 7. It is obvious that there is quite some periodic variation. Also the four data sets show very similar patterns as expected.

**Figure 8:**
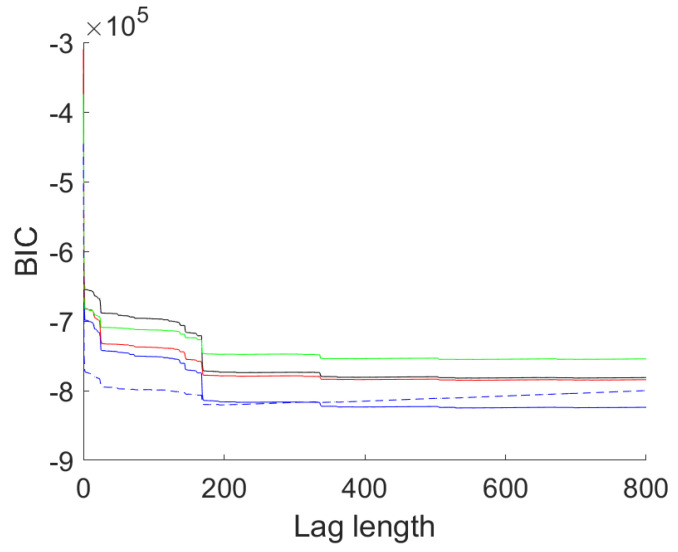
BIC values for univariate models and multivariate model (dashed line; divided by four to fit).

After the extraction of these deterministic terms the next step is univariate autoregressive (AR) modeling. Figure 8 shows the BIC values of AR models of lag lengths zero to 800 for the four series as well as the BIC of a multivariate AR model for the same number of lags. The chosen values are given in Table 6.

The BIC curve is extremely flat for the univariate models. Noticeable drops in BIC occur around lag 24 (one day), 144 (six days), 168 (one week), 336 (two weeks), 504 (three weeks). BIC selects large lag lengths from 529 (DUQ) up to 554 (DOM). AIC selects lag lengths close to the maximum allowed with a minimum at 772 lags. The BIC pattern of the multivariate model differs in that the two drops at two and three weeks are missing. Instead, the optimal BIC value is obtained at lag 194, well below the optimal lag lengths in the univariate cases. AIC here opts for lag length 531, just over 22 days.

Subsequently CVA is applied with $f={\hat{k}}_{BIC},p={\hat{k}}_{AIC}$ as estimated for the multivariate model. This differs from the usual recommendation of $f=p=2{\hat{k}}_{AIC}$ in order to avoid numerical problems with huge matrices. The order is chosen according to SVC, resulting in $\hat{n}=240$. The corresponding model is termed Mod 1 in the following. Note that this configuration of $f,\hat{n}$ does not fulfill the requirements of our asymptotic theory. The bound f≥n ensures that the matrix $O_{f}$ has full column rank. Generically this will be the case for fs≥n leading to a less restrictive assumption. In practice too low values of *f* will be detected by $\hat{n}$ estimated close to the maximum, which is not the case here.

As a second model we only use weekday dummies but neglect the other deterministics. Again AIC (${\hat{k}}_{AIC}=531$) and BIC (${\hat{k}}_{BIC}=195$) are used to determine the optimal lag length in the multivariate AR model. The corresponding CVA estimated model uses $\hat{n}=245$ according to SVC, resulting in Mod 2.

The third model uses only a constant as deterministic term. Again similar AIC (555) and BIC (195) values are selected. A state space model, Mod 3, using CVA is estimated with $\hat{n}=209$.

**Figure 9:**
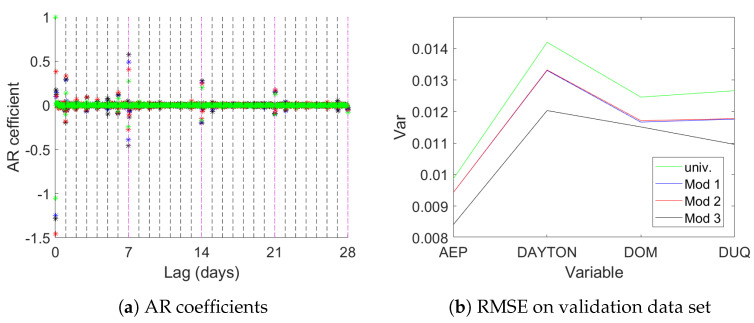
Results for the hourly datasets.

Figure 9 provides information on the results. Panel (a) shows the coefficients of the univariate AR models. It can be seen that lags around one day and one to three weeks play the biggest role for all four datasets. Panel (b) shows that the multivariate models lead to better one step ahead predictions in terms of the root mean square error (RMSE). Mod 1 and Mod 2 show practically equivalent out of sample prediction error for all four data sets, while Mod 3 delivers the best out of sample fit for all four regions.

**Figure 10:**
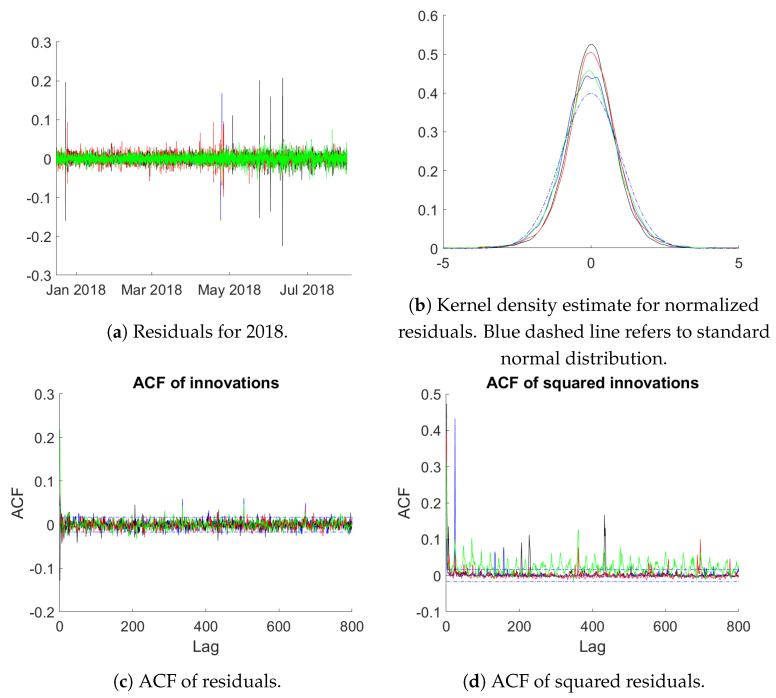
Residual analysis.

In particular in financial applications data of high sampling frequency shows persistent behaviour, also in terms of conditional heteroskedasticity, as well as heavy tailed distributions of the innovations. For our data sets Figure 10 below provides some information in this respect for the residuals according to Mod 3. Panel (a) provides a plot of the residuals in the year 2018 (contained in the validation period). It can be seen that large deviations occur occasionally, while else residuals vary in a tight band around 0. The kernel density estimates for the normalized (to unit variance) residuals on the full validation data set in panel (b) show the typical heavy tailed distributions. Panel (c) contains an ACF plot for the four regions again calculated using the full validation sample. It demonstrates that the model successfully eliminates all autocorrelations with only a few ACF values occurring outside the confidence interval. Panel (d) provides the ACF plot for the squared innovations to examine GARCH-type effects. While GARCH-effects are clearly visible, the ACF drops to zero fast with occasional positive values (except maybe for the Duquesne data).

Applying the eigenvalue based test Λ(1) for c=1 and all Fourier frequencies $\omega_{j}=2\pi j/\left(365*24\right)$ we find that for Mod 2 and Mod 3 the largest *p*-value is obtained for $\omega_{365}$ corresponding to a period length of one day with 0.0187 for Mod 2 (test statistic 6.6) and 0.02 for Mod 3 (with a statistic of 6.5). This implies that the unit root at frequency $\omega_{365}$ is not rejected for a significance level of 1%, but is rejected for 5%. All other unit roots are rejected at every usual significance level. For Mod 1 the test statistic for $\omega_{365}$ equals 41.2 corresponding to a *p*-value of practically 0. This implies that on top of a deterministic daily pattern the series show strong persistence at the daily period. Excluding the hourly dummies pulls the roots closest to $\omega_{365}$ closer to the unit circle resulting in insignificant unit root tests and improves the one step ahead forecasts. Including the dummies weakens the evidence of a unit root while leading to worse predictions.

**Figure 11:**
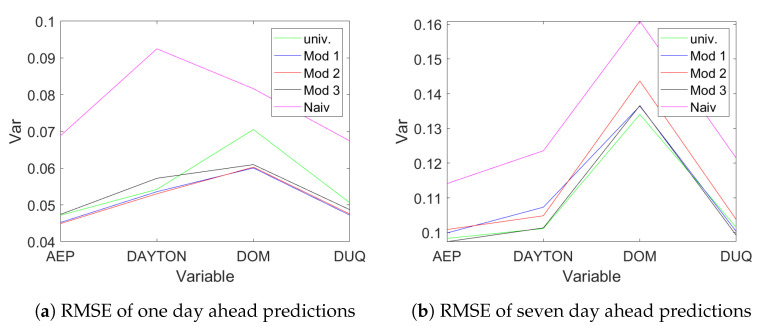
Results for the hourly datasets.

The analysis is repeated with data aggregated to daily sampling frequency. The aggregation reduces the required lag lengths, as is visible from Table 6 in the univariate case, and hence we use CVA with the recommended $f=p=2{\hat{k}}_{AIC}$. Beside the univariate models, in this case also a naive model of predicting the consumption for today as yesterday’s consumption is used. Three multivariate models are estimated: Mod 1 contains weekday dummies and sine and cosine terms for the first twenty Fourier frequencies corresponding to a period of one year. Mod 2 only contains the weekday dummies, while Mod 3 only uses the constant. Figure 11 provides the out-of-sample RMSE for one day ahead predictions (panel (a)) and seven days ahead predictions (panel (b)).

**Figure 12:**
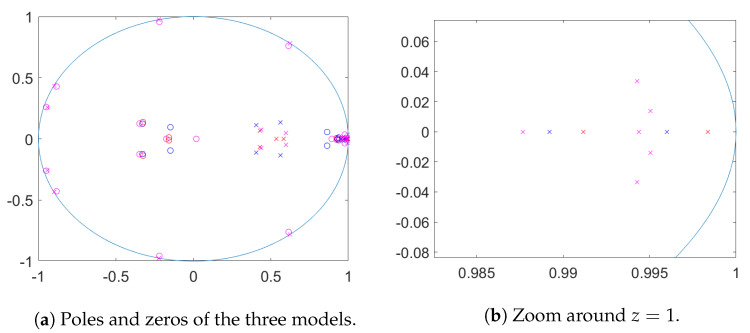
Poles (x) and zeros (o) of the transfer functions corresponding to the three models: Mod 1 (red), Mod 2 (blue), Mod 3 (magenta).

It can be seen that both Mod 1 and Mod 2 beat the univariate AR models in terms of one step ahead prediction error, while Mod 3 performs better for seven days ahead prediction. Mod 1 performs on par with Mod 2 for one step ahead prediction but performs better in predicting seven steps ahead. In Figure 12 poles and zeros for the three estimated state space models are plotted. Here the poles (marked with ‘x’) are the eigenvalues of the matrix *A*. These are the inverses of the determinantal roots of the autoregressive matrix polynomial in the equivalent VARMA representation. The zeros are the inverses of zeros of the determinant of the MA polynomial. We can see that for Mod 3 with only a constant, poles close to 2πj/7,j=1,...,6 arise to capture the weekly pattern. The other two models only show one pole close to the unit circle, a real pole of almost z=1. The pole corresponding to Mod 1 is closer to the unit circle than the one for Mod 2 (see (b)).

For Mod 3 we obtain *p*-values for the tests of three complex unit roots of 0.05 (ω=2π/7), 0.165 (4π/7) and 0.01 (6π/7), which are hence all not statistically significant for significance level α=0.01. The corresponding test for z=1 shows a *p*-value of 0.004. This provides evidence against the null hypothesis of the root being present. For Mod 1 the *p*-value for the test of z=1 is 0.28 and hence we cannot reject the null. Mod 2 provides a *p*-value of 0.023 and hence weak evidence for the presence of the unit root. This can be seen from the distance of the nearest pole from the point z=1 in Figure 12.

Jointly this indicates that the location and strength of persistence due to the estimated roots is influenced by the presence of deterministic terms: if the deterministic terms are not included in the model, the cyclical patterns are generated by poles situated close to the unit circle.

The decision whether on top of the deterministic seasonality unit roots exist, is not easy in all cases: for the daily data the locations of the poles indicate that deterministic seasonality is enough to capture weekly fluctuations while a unit root at z=1 appears to be needed to capture yearly variations. For hourly data there is evidence that the daily cycle is best captured with a unit root at frequency $\omega_{365}$. This leads to the best predictive fit. Finally note that temporal aggregation from hourly data to daily data implies that the frequency $\omega_{365}$ for hourly data aliases to the frequency ω=0 in the daily data. Therefore the higher evidence of a unit root at z=1 found in daily data might be a consequence of the unit root at frequency $\omega_{365}$ found for hourly data, compare [37].

The system matrix estimates as well as the evidence in support of unit roots at $\omega_{365}$ for hourly data and z=1 for daily data that we obtain from the CVA modeling can be taken as starting points in subsequent quasi maximum likelihood estimation.

## 9. Conclusions

In this paper the asymptotic properties of CVA estimators for seasonally integrated unit root processes are investigated. The main results can be summarized as follows:

- CVA provides consistent estimators for long-run and short-run dynamics without knowledge of the location and number of unit roots. Hence the algorithm is robust with respect to the presence of trending components at frequency zero as well as at the other seasonal unit root frequencies.
- The singular values calculated in the RRR step reveal information on the total number of unit roots. The distance of the singular values to one can be used to construct a consistent estimator of this quantity.
- The eigenvalues of $\hat{A}$ can be used in order to test for the number of common trends. Under the null hypothesis these tests are asymptotically equivalent to the corresponding tests using the true state, making the derivation of asymptotic results and the simulation of the test distribution simple.
- An analogous statement holds for the Johansen trace test in the I(1) case and analogous tests in the MFI(1) case calculated on the basis of the estimated state in the restrictive setting of n≤s. Under the null hypothesis these tests reject and accept asymptotically jointly with the corresponding tests calculated using the true state.
- From the simulation exercises we conclude that CVA performs best when the dgp is of the more general VARMA type, the process dimension is moderate to large and the sample size is small. Then it is superior to the likelihood-based procedures based on VAR approximations in terms of the estimation performance and the size and power of Λ, the test developed from CVA. For higher sample sizes the likelihood-based procedures are clearly superior when it comes to the size of the corresponding tests, whereas Λ remains the best test choice in terms of empirical power. The estimation performance is about equal for all procedures when the sample size is high with slight advantages for the likelihood-based procedures.
- The simulations also demonstrate that the unit root test results are robust with respect to the distribution of the innovation sequence as well as some forms of conditional heteroskedasticity of the GARCH-type.

Because of the promising performance of CVA and in particular its robustness it can be recommended as a simple way to extract information on the number of common trends from the estimated matrix of transition dynamics. This information can be used in order to reduce the uncertainty in a subsequent likelihood ratio analysis where quasi maximum likelihood estimates can be obtained starting from the CVA estimates. Since the CVA estimates can be obtained for a range of orders numerically fast they are seen as a valuable starting point for the empirical modeling of time series potentially including seasonal cointegration. Moreover they can also be used in situations where the number of seasons is large or even unclear as in hourly data sets as demonstrated in the case study.   

## Figures and Tables

**Table 1:** Eigenvalues of the coefficient matrix of the companion form.

		j							
		1	2	3	4	5	6	7	8
γ=0.2	$z_{j}$	−1	1	i	−i	0.126 + i0.99	0.126 − i0.99	−0.790	0.737
	$|z_{j}|$	1	1	1	1	0.998	0.998	0.790	0.737
γ=0	$\mu_{j}$	−1	i	−i	1	i	−i	0.775	−0.775
	$|\mu_{j}|$	1	1	1	1	1	1	0.775	0.775

**Table 2:** Hit rates for the different tests (VAR dgp). Twice the maximum (over all entries) Monte Carlo standard error is 0.005.

		0		π/2					π	
	T	Λ	J	Λ	JS	Q1	Q2	Q3	Λ	J
γ=0	50	0.685	0.348	0.351	0.903	0.844	0.851	0.844	0.681	0.343
	100	0.841	0.732	0.490	0.925	0.900	0.902	0.900	0.831	0.724
	200	0.897	0.951	0.841	0.934	0.925	0.924	0.925	0.876	0.936
	500	0.931	0.938	0.916	0.949	0.941	0.942	0.941	0.927	0.948
γ=0.2	50	0.550	0.367	0.811	0.796	0.777	0.778	0.788	0.604	0.297
	100	0.711	0.801	0.087	0.920	0.913	0.908	0.908	0.799	0.806
	200	0.907	0.922	0.855	0.954	0.949	0.948	0.947	0.854	0.939
	500	0.944	0.953	0.927	0.939	0.938	0.938	0.936	0.924	0.942

**Table 3:** Hit rates for the different tests (VARMA dgp). Twice the maximum (over all entries) Monte Carlo standard error is 0.005.

		0		π/2					π	
	T	Λ	J	Λ	JS	Q1	Q2	Q3	Λ	J
$A_{1}$	50	0.890	0.003	0.906	0.024	0.027	0.032	0.025	0.897	0.008
	100	0.928	0.434	0.944	0.755	0.783	0.783	0.761	0.930	0.440
	200	0.936	0.937	0.923	0.925	0.915	0.916	0.915	0.925	0.924
	500	0.852	0.901	0.853	0.919	0.906	0.904	0.904	0.853	0.894
$A_{2}$	50	0.863	0.008	0.785	0.062	0.047	0.063	0.039	0.867	0.006
	100	0.917	0.500	0.880	0.582	0.596	0.596	0.571	0.916	0.518
	200	0.931	0.927	0.882	0.908	0.915	0.913	0.911	0.919	0.922
	500	0.824	0.882	0.786	0.878	0.860	0.859	0.861	0.812	0.865

**Table 4:** Mean gaps between estimated and true cointegrating spaces (VARMA dgp). 2MCse denotes twice the maximal Monte Carlo standard error for the corresponding row.

			0			π/2				π		
	T	2MCse	CVA	J	GARR	CVA	JS	cRRR	GARR	CVA	J	GARR
$A_{1}$	50	0.016	0.116	0.189	0.192	0.091	0.147	0.130	0.130	0.111	0.192	0.197
	100	0.004	0.047	0.048	0.048	0.039	0.035	0.035	0.035	0.047	0.046	0.046
	200	0.003	0.023	0.019	0.019	0.019	0.016	0.016	0.016	0.024	0.019	0.019
	500	0.003	0.012	0.007	0.007	0.008	0.008	0.006	0.006	0.011	0.007	0.007
$A_{2}$	50	0.016	0.174	0.245	0.242	0.250	0.349	0.331	0.331	0.165	0.231	0.234
	100	0.004	0.072	0.061	0.061	0.098	0.080	0.078	0.078	0.069	0.060	0.060
	200	0.003	0.031	0.026	0.026	0.047	0.036	0.034	0.034	0.032	0.027	0.027
	500	0.003	0.016	0.011	0.010	0.021	0.015	0.013	0.013	0.017	0.011	0.011

**Table 5:** Percentage of accept (minimum for all unit root frequencies) and reject (maximum for non unit root frequencies) of Λ(1) test statistic.

	Unit Root Frequencies					Non Unit Root Frequencies				
T	norm	G1	IG1	IG2	IG3	norm	G1	IG1	IG2	IG3
104	0.94	0.89	0.87	0.88	0.87	0.87	0.82	0.79	0.82	0.79
208	0.98	0.96	0.95	0.94	0.96	0.78	0.75	0.72	0.72	0.69
312	0.97	0.96	0.96	0.95	0.95	0.00	0.00	0.00	0.00	0.00

**Table 6:** Summary of data sets.

Region	Daily Obs. (4263 est., 577 val.)					Hourly Obs. (102,291 est., 13,845 val.)			
	Mean	Mean(log)	Std.(log)	AIC	BIC	Mean(log)	Std.(log)	AIC	BIC
AEP	371,844	12.82	0.127	43	12	9.63	0.168	782	532
DAYTON	48,897	10.79	0.144	43	3	7.60	0.193	772	531
DOM	262,727	12.47	0.158	17	3	9.28	0.215	795	554
DUQ	39,837	10.58	0.130	23	7	7.40	0.177	800	529

## Appendix A. Supporting Material

### Appendix A.1. Complex Valued Canonical Form

Additionally to the real valued canonical form (2) we will also use the corresponding complex valued representation obtained by transforming each block corresponding to unit root $z_{j}=\cos\left(\omega_{j}\right)+i\sin\left(\omega_{j}\right)$ with the transformation matrix

$$T_{j}=\left[\begin{array}{cc} I_{c_{j}} & iI_{c_{j}}\\ I_{c_{j}} & -iI_{c_{j}} \end{array}\right]$$

leading to the triple of system matrices in the *j*-th block as:

$$A_{j,C}=\left[\begin{array}{cc} \bar{z_{j}}I_{c_{j}} & 0\\ 0 & z_{j}I_{c_{j}} \end{array}\right],\;K_{j,C}=\left[\begin{array}{c} K_{j,C}\\ \bar{K_{j,C}} \end{array}\right],{\;}_{j,C}=\left[\begin{array}{cc} C_{j,C}/2 & \bar{C_{j,C}}/2 \end{array}\right],$$

such that

$$x_{t+1,j,C}=\bar{z_{j}}x_{t,j,C}+K_{j,C}\varepsilon_{t},\;x_{t,j}=T_{j}^{-1}\left[\begin{array}{c} x_{t,j,C}\\ \bar{x_{t,j,C}} \end{array}\right].$$

**Lemma** **A1.**
*Let $x_{t}={\left[x_{t,0}^{\prime },x_{t,1}^{\prime },\cdots ,x_{t,S/2}^{\prime },x_{t,•}^{\prime }\right]}^{\prime }$ where $x_{t,j}$ is generated according to $x_{t+1,j}=A_{j}x_{t,j}+K_{j}\varepsilon_{t},t\in N$ with $A_{j}$ as in *(2)* and $K_{j}={\left[K_{j,R}^{\prime },K_{j,I}^{\prime }\right]}^{\prime }\in R^{\delta_{j}c_{j}\times s}$ using iid white noise process ${\left(\varepsilon_{t}\right)}_{t\in N}$ where $x_{0,j}$ is deterministic. Further let ${\left(x_{t,•}\right)}_{t\in N}$ denote the stationary solution to the equation $x_{t+1,•}=A_{•}x_{t,•}+K_{•}\varepsilon_{t}$ such that $M_{•}=Ex_{t,•}x_{t,•}^{\prime }>0$.*

*(I) Then using $Q_{T}=\sqrt{(\log\log T)/T}$ for $u_{t}=\sum_{i=0}^{q}\varphi_{i}\varepsilon_{t+i}$ for arbitrary q∈N,q<∞, and coefficients $\varphi_{i},i=0,...,q$ we have*

$$\begin{array}{ccc} \langle x_{t,•},u_{t}\rangle =O\left(Q_{T}\right) & , & \langle u_{t-j},u_{t}\rangle -Eu_{t-j}u_{t}^{\prime }=O\left(Q_{T}\right),\\ \langle x_{t,j},x_{t,•}\rangle =O\left(\log T\right) & , & \langle x_{t,j},u_{t}\rangle =O\left(\log T\right)\\ \langle x_{t,j},x_{t,k}\rangle /T=O\left(\log\log T\right) & , & j,k=0,...,S/2. \end{array}$$

*If ${\left(\varepsilon_{t}\right)}_{t\in Z}$ only fulfills Assumptions 1 then the order bounds hold in probability rather than almost surely.*

*(II) Furthermore for 0<j,k<S/2*

$$\begin{array}{ccc} \langle x_{t,j,C},\varepsilon_{t}\rangle & \overset{d}{\to } & \frac{1}{2}\int_{0}^{1}W_{j}dB_{j,C}^{\prime }=:M_{j},\\ \langle x_{t,j,C},x_{t,k,C}\rangle /T & \overset{d}{\to } & \left\{\begin{array}{ccc} \frac{1}{2}\int_{0}^{1}W_{j}W_{j}^{\prime }:=N_{j} & , & j=k,\\ 0 & , & j\neq k \end{array}\right.\\ \langle x_{t,j},\varepsilon_{t}\rangle & \overset{d}{\to } & \left[\begin{array}{c} \frac{1}{2}\int_{0}^{1}\left(W_{j,R}dB_{j,R}^{\prime }+W_{j,I}dB_{j,I}^{\prime }\right)\\ \frac{1}{2}\int_{0}^{1}\left(W_{j,I}dB_{j,R}^{\prime }-W_{j,R}dB_{j,I}^{\prime }\right) \end{array}\right],\\ \langle x_{t,k},x_{t,j}\rangle /T & \overset{d}{\to } & \left\{\begin{array}{ccc} \frac{1}{2}\left[\begin{array}{cc} \int_{0}^{1}\left(W_{k,R}W_{k,R}^{\prime }+W_{k,I}W_{k,I}^{\prime }\right) & \int_{0}^{1}\left(W_{k,R}W_{k,I}^{\prime }-W_{k,I}W_{k,R}^{\prime }\right)\\ -\int_{0}^{1}\left(W_{k,R}W_{k,I}^{\prime }-W_{k,I}W_{k,R}^{\prime }\right) & \int_{0}^{1}\left(W_{k,R}W_{k,R}^{\prime }+W_{k,I}W_{k,I}^{\prime }\right) \end{array}\right] & , & j=k\\ 0 & , & j\neq k \end{array}\right. \end{array}$$

*where $W_{j}=W_{j,R}+iW_{j,I}=K_{j,C}B_{j,C},K_{j,C}=K_{j,R}+iK_{j,I},B_{j,C}=B_{j,R}+iB_{j,I}$ and $B_{j,R},B_{j,I}$ are two independent Brownian motions with covariance matrix *Ω*. For j=0 and j=S/2 the results hold analogously:*

$$\begin{array}{ccc} \langle x_{t,0},\varepsilon_{t}\rangle \overset{d}{\to }\int_{0}^{1}W_{0,R}dW_{0,R}^{\prime } & , & \langle x_{t,0},x_{t,0}\rangle /T\overset{d}{\to }\int_{0}^{1}W_{0,R}W_{0,R}^{\prime },\\ \langle x_{t,S/2},\varepsilon_{t}\rangle \overset{d}{\to }\int_{0}^{1}W_{S/2,R}dW_{S/2,R}^{\prime } & , & \langle x_{t,S/2},x_{t,S/2}\rangle /T\overset{d}{\to }\int_{0}^{1}W_{S/2,R}W_{S/2,R}^{\prime }. \end{array}$$

**Proof.** Most evaluations in (I) are standard, see for example Lemma 4 in [38]. (II) follows from the results in Section 4 of [2] for the complex valued representations or [39] for the corresponding real case.    □

### Appendix A.2. Perturbation of Eigendecompositions

**Lemma** **A2** (Rayleigh-Schrödinger expansion). *Let ${\hat{A}}_{t}=A-\delta A_{t}$ where $∥\delta A_{t}∥\to 0$ and where $A=U\Lambda U^{-1}\in R^{n\times n},\Lambda =diag\left(\lambda_{1}I_{c_{1}},...,\lambda_{J}I_{c_{J}}\right),\sum_{j=1}^{J}c_{j}=n$ is diagonalizable. $U=\left[U_{1},...,U_{J}\right]\in C^{n\times n}$ is a nonsingular matrix such that for $U^{-1}={\left[V_{1},...,V_{J}\right]}^{\prime }$ we have $V_{j}^{\prime }U_{j}=I_{c_{j}}$.*
*Then for each circle $B(\lambda_{j},\delta )$ around $\lambda_{j}$ not containing any other eigenvalue of A there exist from some t onwards*

- *$c_{j}$ eigenvalues of ${\hat{A}}_{t}$ in the circle $B(\lambda_{j},\delta )$ around $\lambda_{j}$*
- *a basis ${\hat{U}}_{t,j}$ for the space spanned by the eigenspaces to these $c_{j}$ eigenvalues such that $V_{j}^{\prime }{\hat{U}}_{t,j}=I_{c_{j}}$,*
- *a sequence of matrices ${\hat{B}}_{t,j}=V_{j}^{\prime }{\hat{A}}_{t}{\hat{U}}_{t,j}\in C^{c_{j}\times c_{j}}$.*

*Then ${\hat{U}}_{t,j}=\sum_{k=0}^{\infty }Z_{k},{\hat{B}}_{t,j}=\sum_{k=0}^{\infty }C_{k}$ where*

$$\begin{array}{ccc} Z_{0}=U_{j} & , & C_{0}=\lambda_{j}I_{c_{j}},\\ Z_{k}=\Sigma \left(\delta A_{t}Z_{k-1}+\sum_{i=1}^{k-1}Z_{k-i}C_{i}\right) & , & C_{k}=-V_{j}^{\prime }\delta A_{t}Z_{k-1}. \end{array}$$

*Here $\Sigma =U{\left(\Lambda -I_{n}\lambda_{j}\right)}^{+}U^{-1}$ where $diag{\left(s_{1},...,s_{n}\right)}^{+}=diag\left(s_{1}^{+},...,s_{n}^{+}\right)$ and $x^{+}=1/x,x\neq 0$ and zero else, that is ${\left(\Lambda -I_{n}\lambda_{j}\right)}^{+}$ denotes a quasi-inverse.*

*Furthermore for $\rho =∥\delta A_{t}∥<1$ we obtain: $∥C_{k}∥\leq \mu_{C}\rho^{k},∥Z_{k}∥\leq \mu_{Z}\rho^{k},\;k\geq 0$.*

The results follow directly from Section 2.9 of [23], see in particular Proposition 2.9.1 and the discussion below this proposition. Further note that the results hold for each root separately and hence the restriction $\ell_{j}=1$ needs to hold only for the investigated root for the results to apply. Finally note that a second order approximation ${\hat{U}}_{t,j}=Z_{0}+Z_{1}+Z_{2}$ and ${\hat{B}}_{t,j}=C_{0}+C_{1}+C_{2}$ is accurate to the order $o(∥\delta A_{t}{∥}^{2})$.

### Appendix A.3. Random Transformation of Systems

**Lemma** **A3.**
*Let the assumptions of Theorem 1 hold and use the same notation as given there. Let $\left(\tilde{A},\tilde{C},\tilde{K}\right)$ denote a sequence of systems converging a.s. to (A,C,K) such that $\left(\tilde{A}-A\right)D_{x}^{-1}=O\left({\left(\log T\right)}^{a}\right),\sqrt{T}\left(\tilde{K}-K\right)=O\left({\left(\log T\right)}^{a}\right),\left(\tilde{C}-C\right)D_{x}^{-1}=O\left({\left(\log T\right)}^{a}\right)$ and let $A_{0}=S_{0}AS_{0}^{-1}=diag\left(A_{0,11},A_{0,22}\right),K_{0}=S_{0}K,C_{0}=CS_{0}^{-1}$. Further let*

$$S_{T}=\left[\begin{array}{cc} S_{T,11} & S_{T,12}\\ 0 & S_{T,22} \end{array}\right]\to S_{0}$$

*such that $\left(S_{T}-S_{0}\right)D_{x}^{-1}=O\left({\left(\log T\right)}^{a}\right)$. Let $\Delta S=\left(S_{T}-S_{0}\right)D_{x}^{-1},\Delta A=\left(\tilde{A}-A\right)D_{x}^{-1}$ and denote the sequence of transformed systems as $\left(\hat{A},\hat{C},\hat{K}\right)=\left(S_{T}\tilde{A}S_{T}^{-1},\tilde{C}S_{T}^{-1},S_{T}\tilde{K}\right)$. Let the block entries of $S_{0}$ be denoted as $S_{ij}$ and the blocks of ΔS be denoted as $\Delta S_{ij}$. Then:*

$$\begin{array}{ccc} T({\hat{A}}_{11}-A_{0,11}) & = & \left(\Delta S_{11}A_{11}-A_{0,11}\Delta S_{11}+S_{11}\Delta A_{11}+S_{12}\Delta A_{21}\right)S_{11}^{-1}+o\left(1\right),\\ \sqrt{T}\left({\hat{A}}_{12}-A_{0,12}\right) & = & \left(S_{11}\Delta A_{12}+S_{12}\Delta A_{22}\right)S_{22}^{-1}+\Delta S_{12}S_{22}^{-1}A_{0,22}-A_{0,11}\Delta S_{12}S_{22}^{-1}+o\left(1\right),\\ T({\hat{A}}_{21}-A_{0,21}) & = & S_{22}\Delta A_{21}S_{11}^{-1}+o\left(1\right),\\ \sqrt{T}\left({\hat{A}}_{22}-A_{0,22}\right) & = & \Delta S_{22}S_{22}^{-1}A_{0,22}+S_{22}\Delta A_{22}S_{22}^{-1}-A_{0,22}\Delta S_{22}S_{22}^{-1}+o\left(1\right),\\ \sqrt{T}\left(\hat{K}-K_{0}\right) & = & \left[\begin{array}{c} \Delta S_{12}K_{2}+S_{11}\sqrt{T}\left({\tilde{K}}_{1}-K_{1}\right)+S_{12}\sqrt{T}\left({\tilde{K}}_{2}-K_{2}\right)\\ \Delta S_{22}K_{2}+S_{22}\sqrt{T}\left({\tilde{K}}_{2}-K_{2}\right) \end{array}\right]+o\left(1\right),\\ \left(\hat{C}-C_{0}\right)D_{x}^{-1} & = & \left(\hat{C}-C\right)D_{x}^{-1}\left[\begin{array}{cc} S_{11}^{-1} & 0\\ 0 & S_{22}^{-1} \end{array}\right]-C_{0}\left[\begin{array}{cc} \Delta S_{11}S_{11}^{-1} & \Delta S_{12}S_{22}^{-1}\\ 0 & \Delta S_{22}S_{22}^{-1} \end{array}\right]+o\left(1\right). \end{array}$$

**Proof.** The proof follows from straightforward computations using the various orders of convergence by neglecting higher order terms.    □

## Appendix B. Reduced Rank Regression with Integrated Variables

The main results of this paper are based on a more general result documented in [24] (henceforth called BRRR). BRRR uses a slightly different setting and in particular a different dgp. The following lemma provides the essence of the results of BRRR that will be used below.

**Lemma** **A4.**
*Let ${\left(y_{t}\right)}_{t\in N},{\left(z_{t}^{r}\right)}_{t\in N},{\left(z_{t}^{u}\right)}_{t\in N},y_{t}\in R^{s},z_{t}^{r}\in R^{m},z_{t}^{u}\in R^{l}$ be three processes related via*

$$y_{t}=b_{r}z_{t}^{r}+b_{u}z_{t}^{u}+u_{t}$$

*where the zero mean stationary process ${\left(u_{t}\right)}_{t\in N}$ is such that $Eu_{t}{\left(z_{t}^{r}\right)}^{\prime }=0,Eu_{t}{\left(z_{t}^{u}\right)}^{\prime }=0,Eu_{t}u_{t}^{\prime }>0$ and where n= rank $\left(b_{r}\right)<\min\left(s,m\right)$, that is $b_{r}$ is of reduced rank.*

*Further assume that there exist square nonsingular matrices $T_{y}\in R^{s\times s},T_{r}\in R^{m\times m},T_{u}\in R^{n\times n}$ such that*

$${\tilde{y}}_{t}=T_{y}y_{t}=\left(T_{y}b_{r}T_{r}^{-1}\right)\left(T_{r}z_{t}^{r}\right)+\left(T_{y}b_{r}T_{r}^{-1}\right)\left(T_{r}z_{t}^{r}\right)+T_{y}u_{t}={\tilde{b}}_{r}{\tilde{z}}_{t}+{\tilde{b}}_{u}{\tilde{z}}_{t}^{u}+{\tilde{u}}_{t}$$

*such that with $c_{•}=n-c$ we have*

$${\tilde{b}}_{r}=\left[\begin{array}{ccc} I_{c} & 0 & 0\\ 0 & 0 & {\tilde{b}}_{r,•} \end{array}\right],\;{\tilde{b}}_{r,•}={\tilde{O}}_{•}\Gamma_{•}^{\prime },\;{\tilde{O}}_{•}\in R^{\left(s-c\right)\times c_{•}},\;\Gamma_{•}\in R^{m_{•}\times c_{•}}.$$

*Here the partitioning corresponds to ${\tilde{z}}_{t}^{\prime }=\left[{\tilde{z}}_{t,1}^{\prime },{\tilde{z}}_{t,2}^{\prime },{\tilde{z}}_{t,•}^{\prime }\right]$ where ${\tilde{z}}_{t,1}\in R^{c},{\tilde{z}}_{t,2}\in R^{m-c-m_{•}}$ are MFI(1) processes and ${\left({\tilde{z}}_{t,•}\right)}_{t\in N},{\tilde{z}}_{t,•}\in R^{m_{•}}$ is stationary, ${\tilde{z}}_{t}^{u}={\left[{\left({\tilde{z}}_{t,1}^{u}\right)}^{\prime },{\left({\tilde{z}}_{t,•}^{u}\right)}^{\prime }\right]}^{\prime }$ where ${\left({\tilde{z}}_{t,1}^{u}\right)}_{t\in N}$ is a MFI(1) process and ${\left({\tilde{z}}_{t,•}^{u}\right)}_{t\in N}$ is stationary and where the following bounds hold (${\tilde{z}}_{t,:}={\left[{\tilde{z}}_{t,1}^{\prime },{\tilde{z}}_{t,2}^{\prime }\right]}^{\prime }$):*

$$\begin{array}{ccccc} \langle {\tilde{u}}_{t},{\tilde{u}}_{t}\rangle =O\left(1\right) & , & \langle {\tilde{u}}_{t},{\tilde{z}}_{t,•}\rangle =O\left(Q_{T}\right) & , & \langle {\tilde{u}}_{t},{\tilde{z}}_{t,•}^{u}\rangle =O\left(Q_{T}\right),\\ \langle {\tilde{u}}_{t},{\tilde{u}}_{t}\rangle -E{\tilde{u}}_{t}{\tilde{u}}_{t}^{\prime }=O\left(Q_{T}\right) & , & \langle {\tilde{u}}_{t},{\tilde{z}}_{t,:}\rangle =O\left(\log T\right) & , & \langle {\tilde{u}}_{t},{\tilde{z}}_{t,1}^{u}\rangle =O\left(\log T\right),\\ {\hat{M}}_{•}=\langle \left(\begin{array}{c} {\tilde{z}}_{t,•}\\ {\tilde{z}}_{t,•}^{u} \end{array}\right),\left(\begin{array}{c} {\tilde{z}}_{t,•}\\ {\tilde{z}}_{t,•}^{u} \end{array}\right)\rangle & , & {\hat{M}}_{•}^{-1}=O\left(1\right) & , & {\hat{M}}_{•}=O\left(1\right),M_{•}>0\\ {\hat{M}}_{1}=\langle \left(\begin{array}{c} {\tilde{z}}_{t,:}\\ {\tilde{z}}_{t,1}^{u} \end{array}\right),\left(\begin{array}{c} {\tilde{z}}_{t,:}\\ {\tilde{z}}_{t,1}^{u} \end{array}\right)\rangle & , & {\hat{M}}_{1}/T=O\left(\log\log T\right) & , & {\left({\hat{M}}_{1}\right)}^{-1}=O\left(Q_{T}^{2}\right),\\ \langle \left(\begin{array}{c} {\tilde{z}}_{t,•}\\ {\tilde{z}}_{t,•}^{u} \end{array}\right),\left(\begin{array}{c} {\tilde{z}}_{t,:}\\ {\tilde{z}}_{t,1}^{u} \end{array}\right)\rangle =O\left(\log T\right) & , & {\hat{M}}_{•}-M_{•}=O\left(Q_{T}\right). \end{array}$$

*Then the reduced rank regression estimator ${\hat{b}}_{RRR}=\left[{\hat{b}}_{r,RRR},{\hat{b}}_{u,RRR}\right]$ maximizing the Gaussian likelihood subject to rank $\left(\beta_{r}\right)=n=c+c_{•}$ is consistent: ${\hat{b}}_{RRR}-b=O\left({\left(\log T\right)}^{a}/\sqrt{T}\right)$ for some a<∞. Furthermore ${\tilde{b}}_{RRR,r}-{\tilde{b}}_{r}=\left[O\left({\left(\log T\right)}^{a}/T\right),O\left({\left(\log T\right)}^{a}/\sqrt{T}\right)\right]$ with ${\tilde{b}}_{RRR,r}=T_{y}{\hat{b}}_{RRR,r}T_{r}^{-1}$, where the second block has $m_{•}$ columns and corresponds to the stationary components of the regressor vector.*

**Proof.** The theorem slightly extends the results of BRRR by adding high level assumptions instead of low level assumptions on the data generating process. The proof hence consists in adjusting the proof in BRRR. In the following we only indicate where arguments in BRRR need to be replaced. A detailed proof would replicate much of the arguments in BRRR and hence is omitted. The representation of Theorem 3.1 in BRRR is contained in the assumptions. Then consistency follows from examining the proof of the first part of Theorem 3.2 in BRRR: essential for the norm bounds are Lemma A.1 (I) and (III). The norm bounds stated under point (I) are directly assumed in this lemma except for the filtered version using $n_{t}$ in place of $x_{t}$. Instead, here the results for $n_{t}$ which are needed in the proof of Theorem 3.2 of BRRR are directly assumed. (III) then follows. Lemmas A.3–A.5 in BRRR do not depend on the assumptions on the various processes and hence continue to hold. Then the proof for consistency in Appendix A.3.1 of BRRR only uses these norm bounds referring also to [38] (which is also only based on the norm bounds contained in the assumptions of this lemma) and hence continues to hold.    □

## Appendix C. Proofs of the Theorems

### Appendix C.1. Proof of Theorem 1

For proving consistency of the transfer function estimators it is sufficient to find a (possibly) random matrix ${\tilde{S}}_{T}$ such that the least squares estimates $\left(\tilde{A},\tilde{C},\tilde{K}\right)$ of one representation (A,C,K) of the true system obtained using ${\tilde{x}}_{t}:={\tilde{S}}_{T}{\hat{x}}_{t}$ converges (a.s.) to (A,C,K). This will be done in two steps: First a particular basis (which is not realizable in practice) will be chosen such that ${\tilde{K}}_{p}-K_{p}=o\left(1\right)$ sufficiently fast such that in the second step the regressions in the system equations based on the resulting state estimator ${\tilde{x}}_{t}$ are consistent. The derivation of the first step will also provide an approximation of the error term which can be used in order to derive the asymptotic distribution.

#### Appendix C.1.1. Proof of Theorem 1 (I)

The central step in CVA is the solution to the RRR problem. The following proof heavily draws on the results contained in [24] (henceforth called BRRR) collected in Lemma A4 for easier reference. As in BRRR, in order to derive the asymptotic properties we first transform the vectors in order to separate stationary and nonstationary terms. In order to achieve the separation let $Z_{t}={\left[y_{t-1}^{\prime },y_{t-2}^{\prime },...,y_{t-S}^{\prime }\right]}^{\prime }\in R^{sS}$. Then for p=kS we obtain

$$Y_{t,p}^{-}=\left(\begin{array}{c} y_{t-1}\\ y_{t-2}\\ \vdots \\ y_{t-S}\\ y_{t-S-1}\\ \vdots \\ y_{t-kS} \end{array}\right)=\left(\begin{array}{c} Z_{t}\\ Z_{t-S}\\ \vdots \\ Z_{t-(k-1)S} \end{array}\right).$$

It is easy to see that for each *j* the process ${\left(Z_{rS-j}\right)}_{r\in N}$ is an I(1) process. Moreover the strict minimum-phase condition for $\left(A_{\circ },C_{\circ },K_{\circ }\right)$ implies that also for the system corresponding to ${\left(Z_{rS-j}\right)}_{r\in N}$ the strict minimum-phase condition holds.

Define the transformation $T_{S}:={\left[O_{S,1},O_{S,⊥}\right]}^{\prime }$ where $O_{S,1}\in R^{sS\times c}$ denotes the matrix containing the first *c* columns of $O_{S}$ for the system $\left(A_{\circ },C_{\circ },K_{\circ }\right)$ in the canonical form. Further $O_{S,⊥}$ is a block column of an orthonormal matrix such that $O_{S,⊥}^{\prime }O_{S,1}=0$. Then the argument of [20] shows that in $T_{S}Z_{t}$ the first *c* components are integrated while the remaining sS−c components are stationary. Then consider for p=kS<t≤T−f+1 (using $O_{S,1}^{†}={\left(O_{S,1}^{\prime }O_{S,1}\right)}^{-1}O_{S,1}^{\prime }$)

(A1) $${\tilde{z}}_{t,p}:=\left[\begin{array}{c} O_{S,1}^{†}O_{S}\left(x_{t}-{\bar{A}}_{\circ }^{p}x_{t-p}\right)\\ O_{S,⊥}^{\prime }Z_{t}\\ O_{S,1}^{†}\left(Z_{t}-Z_{t-S}\right)\\ O_{S,⊥}^{\prime }Z_{t-S}\\ \vdots \\ O_{S,1}^{†}\left(Z_{t-(k-2)S}-Z_{t-(k-1)S}\right)\\ O_{S,⊥}^{\prime }Z_{t-(k-1)S} \end{array}\right],\;{\tilde{y}}_{t}:=\left[\begin{array}{c} O_{f,1}^{†}\\ O_{f,⊥}^{\prime } \end{array}\right]Y_{t,f}^{+}.$$

Here $O_{f,⊥}$ is a matrix such that $O_{f,⊥}^{\prime }O_{f,1}=0,O_{f,⊥}^{\prime }O_{f,⊥}=I$. Obviously ${\tilde{z}}_{t,p}$ is a linear transformation of $Y_{t,p}^{-}$ and ${\tilde{y}}_{t}$ of $Y_{t,f}^{+}$. It can be shown that the linear transformation is nonsingular such that there is a one-one relation between $Y_{t,p}^{-}$ and ${\tilde{z}}_{t,p}$. In ${\tilde{z}}_{t,p}$ and ${\tilde{y}}_{t}$ only the first *c* components are unit root processes, the remaining components being stationary.

For p≠kS the final p−kS block rows of ${\tilde{z}}_{t,p}$ are defined as $y_{t-(k-1)S-j}-y_{t-kS-j},j=1,...,p-kS$. Clearly also these components are stationary.

Partition ${\tilde{z}}_{t,p}={\left[{\tilde{z}}_{t,1}^{\prime },{\tilde{z}}_{t,•}^{\prime }\right]}^{\prime },{\tilde{z}}_{t,1}\in R^{c},$ into its first *c* and the remaining coordinates (omitting the subscript ${}_{p}$ on the right hand side for notational convenience). Similarly partition ${\tilde{y}}_{t}={\left[{\tilde{y}}_{t,1}^{\prime },{\tilde{y}}_{t,•}^{\prime }\right]}^{\prime },{\tilde{y}}_{t,1}\in R^{c}$. Using these transformed matrices, $Y_{t,f}^{+}=\beta_{1}Y_{t,p}^{-}+N_{t,f}^{+}$ can be written as

(A2) $${\tilde{y}}_{t}={\tilde{b}}_{1}{\tilde{z}}_{t,p}+{\tilde{N}}_{t,f,p}^{+}=\left[\begin{array}{c} {\tilde{y}}_{t,1}\\ {\tilde{y}}_{t,•} \end{array}\right]=\left[\begin{array}{cc} I_{c} & 0\\ 0 & {\tilde{b}}_{•,p} \end{array}\right]\left[\begin{array}{c} {\tilde{z}}_{t,1}\\ {\tilde{z}}_{t,•} \end{array}\right]+\tilde{O_{f}}{\bar{A}}_{\circ }^{p}x_{t-p}+\left[\begin{array}{c} {\tilde{\varepsilon }}_{t,1}\\ {\tilde{\varepsilon }}_{t,•} \end{array}\right]$$

where

$${\tilde{b}}_{1}=\left[\begin{array}{cc} I_{c} & 0\\ 0 & {\tilde{b}}_{•,p} \end{array}\right],\;{\tilde{b}}_{•,p}=E{\tilde{y}}_{t,•}{\tilde{z}}_{t,•}^{\prime }{\left(E{\tilde{z}}_{t,•}{\tilde{z}}_{t,•}^{\prime }\right)}^{-1}=O_{•,p}\Gamma_{•,p}^{\prime },\;{\tilde{b}}_{1}=O_{p}\Gamma_{p}^{\prime }$$

and where ${\tilde{b}}_{•,p}$ is of rank n−c providing a representation of the form given in Theorem 3.1 of BRRR except that the error term ${\tilde{N}}_{t,f,p}^{+}$ (defined by the equation) is not white. Finally (A2) also defines the sub blocks ${\tilde{\varepsilon }}_{t,i}$ of ${\tilde{N}}_{t,f,p}^{+}$ which are hence linear combinations of $E_{t,f}^{+}$ and therefore typically MA(f) processes. Note that ${\tilde{z}}_{t,1},{\tilde{z}}_{t,•},{\tilde{y}}_{t,•}$ depend on the choice of *f* and *p* which is not reflected in the notation.

Here ${\left(E{\tilde{z}}_{t,•}{\tilde{z}}_{t,•}^{\prime }\right)}^{-1}$ and $E{\tilde{y}}_{t,•}{\tilde{z}}_{t,•}^{\prime }$ are worth a remark: for p=kS the results of [20] can be directly used to obtain upper and lower bounds for the norms of these matrices uniformly in k∈N. For p≠kS the additional rows in ${\tilde{z}}_{t,•}$ add entries to $E{\tilde{y}}_{t,•}{\tilde{z}}_{t,•}^{\prime }$ that are of order $O(\lambda^{p})$ for some 0<λ<1 as $y_{t}-y_{t-S}$ is a VARMA process. Similarly the smallest eigenvalue of $E{\tilde{z}}_{t,•}{\tilde{z}}_{t,•}^{\prime }$ can be bounded from below based on arguments for p=kS following [20] which in turn refer to Theorem 6.6.10 of [22]. The additional terms for p≠kS correspond to backward innovations with non-singular covariance matrix thus also leading to a lower bound of the smallest eigenvalue uniformly in *k*. (The backward innovations representation for a stationary VARMA process ${\left(y_{t}\right)}_{t\in Z}$ equals $y_{t}=\sum_{j=1}^{\infty }k_{j}^{b}y_{t+j}+\varepsilon_{t}^{b}$ and can be obtained from the complex conjugate of the spectral density. Nonsingularity of the spectral density implies that the backward innovation $\varepsilon_{t}^{b}$ have nonsingular covariance matrix. This implies a lower bound on the accuracy with which components of $y_{t-(k-1)S-j}$ can be predicted based on $y_{t-i},i\leq \left(k-1\right)S$.)

Furthermore the strict minimum-phase assumption for the state space representation $\left(A_{\circ },C_{\circ },K_{\circ }\right)$ of the process ${\left(y_{t}\right)}_{t\in Z}$ implies the strict minimum-phase assumption for the sub-sampled process ${\left(Z_{kS+j}\right)}_{k\in Z}$. Thus the arguments of [20] show that $\left[{\tilde{b}}_{•,p},0\right]\to {\tilde{b}}_{•,\infty }$ where the norm of the difference is of order $O(∥{\bar{A}}_{\circ }^{p}∥)$. The increase of *p* as a function of the sample size jointly with the strict minimum-phase assumption implies that $O(∥{\bar{A}}_{\circ }^{p}∥)=o\left(T^{-1}\right)$. This also implies that $\tilde{O_{f}}{\bar{A}}_{\circ }^{p}x_{t-p}=o_{p}\left(T^{-1/2}\right)$.

Correspondingly there exists a limiting decomposition ${\tilde{b}}_{•,\infty }=O_{•}\Gamma_{•}^{\prime }$ such that $\Gamma_{•}^{\prime }S_{•}=I_{n-c}$ where $S_{•}$ denotes a selector matrix whose columns contain the vectors of the canonical basis of $R^{\infty }$. Since $\left[K_{\circ },\left(A_{\circ }-K_{\circ }C_{\circ }\right)K_{\circ },{\left(A_{\circ }-K_{\circ }C_{\circ }\right)}^{2}K_{\circ },...,{\left(A_{\circ }-K_{\circ }C_{\circ }\right)}^{n-1}K_{\circ }\right]$ is of full row rank it can be assumed that $S_{•}$ only has nonzero entries in its first ns rows. Denoting the submatrix of the first ps rows by $S_{p,2}$ then also ${\left[\Gamma_{•}^{\prime }\right]}_{1:p}S_{p,2}=I_{n-c}$ where ${\left[.\right]}_{1:p}$ denotes the first *p* block columns of a matrix. This fixes a unique decomposition of ${\tilde{b}}_{•}$ and hence $O_{•}$ and $\Gamma_{•}$ do not depend on *p*. Convergence of ${\tilde{b}}_{•,p}$ to ${\tilde{b}}_{•}$ jointly with the lower bound on p(T) then implies convergence of order $o(T^{-1})$ of $O_{•,p}$ to $O_{•}$ and $\Gamma_{•,p}^{\prime }$ to ${\left[\Gamma_{•}^{\prime }\right]}_{1:p}$ using the decomposition of ${\tilde{b}}_{•,p}$ such that $\Gamma_{•,p}^{\prime }S_{p,2}=I_{n-c}$. Correspondingly $O_{p}\to O$ and $∥\Gamma_{p}^{\prime }-{\left[\Gamma^{\prime }\right]}_{1:p}∥\to 0$.

Therefore the reduced rank regression in the CVA procedure shows the same structure as investigated in Lemma A4 with the differences that ${\tilde{z}}_{t,2}$ and ${\tilde{z}}_{t}^{u}$ do not occur, and ${\tilde{z}}_{t,•}$ has increasing size as a function of the sample size. The next lemma therefore extends the results of BRRR to the RRR used in CVA:

In the following we will use a generic a∈N in statements like $O({\left(\log T\right)}^{a})$, not necessarily the same in each occurrence. In this sense e.g., the product of two terms that are $O({\left(\log T\right)}^{a})$ is again taken to be $O({\left(\log T\right)}^{a})$.

**Lemma** **A5.**
*Let the assumptions of Theorem 1 hold where additionally ${\left(\varepsilon_{t}\right)}_{t\in Z}$ is iid. Introduce the notation*

$$\begin{array}{ccc} {\tilde{D}}_{z}=diag\left(T^{-1/2}I_{c},I_{ps-c}\right), & {\tilde{D}}_{y}=diag\left(T^{-1/2}I_{c},I_{fs-c}\right), & {\tilde{D}}_{x}=diag\left(T^{-1/2}I_{c},I_{n-c}\right). \end{array}$$

*Let ${\bar{G}}_{p}$ denote a solution to*

$$\left({\tilde{D}}_{z}\langle {\tilde{z}}_{t,p},{\tilde{y}}_{t}\rangle {\tilde{D}}_{y}\right){\left({\tilde{D}}_{y}\langle {\tilde{y}}_{t},{\tilde{y}}_{t}\rangle {\tilde{D}}_{y}\right)}^{-1}\left({\tilde{D}}_{y}\langle {\tilde{y}}_{t},{\tilde{z}}_{t,p}\rangle {\tilde{D}}_{z}\right){\bar{G}}_{p}=\left({\tilde{D}}_{z}\langle {\tilde{z}}_{t,p},{\tilde{z}}_{t,p}\rangle {\tilde{D}}_{z}\right){\bar{G}}_{p}{\bar{R}}^{2}$$

*using the notation of *(A1)* where ${\bar{R}}^{2}\to \Theta^{2}=diag\left(I_{c},\Theta_{•}\right)\in R^{n\times n}$ and where ${\bar{G}}_{p}$ is normalized such that ${\bar{G}}_{1,1,p}=I_{c},{\bar{G}}_{•,2,p}^{\prime }S_{p,2}=I_{n-c}$ for a selector matrix $S_{p,2}$. Further let*

$${\bar{\Gamma }}_{p}=\left[\begin{array}{cc} I_{c} & 0\\ 0 & {\bar{\Gamma }}_{•,p} \end{array}\right],{\bar{\Gamma }}_{•,p}^{\prime }S_{p,2}=I_{n-c}$$

*denote the solution to the decoupled problem where the stationary and the nonstationary subproblem are separated:*

$$\left(\begin{array}{c} \langle {\tilde{z}}_{t,1},{\tilde{y}}_{t,1}\rangle {\langle {\tilde{y}}_{t,1},{\tilde{y}}_{t,1}\rangle }^{-1}\langle {\tilde{y}}_{t,1},{\tilde{z}}_{t,1}\rangle {\bar{\Gamma }}_{1,1,p}\\ \langle {\tilde{z}}_{t,•},{\tilde{y}}_{t,•}\rangle {\langle {\tilde{y}}_{t,•},{\tilde{y}}_{t,•}\rangle }^{-1}\langle {\tilde{y}}_{t,•},{\tilde{z}}_{t,•}\rangle {\bar{\Gamma }}_{•,p} \end{array}\right)=\left(\begin{array}{c} \langle {\tilde{z}}_{t,1},{\tilde{z}}_{t,1}\rangle {\bar{\Gamma }}_{1,1,p}{\bar{\Theta }}_{1}\\ \langle {\tilde{z}}_{t,•},{\tilde{z}}_{t,•}\rangle {\bar{\Gamma }}_{•,p}{\bar{\Theta }}_{•} \end{array}\right).$$

*(I) Then if f≥n fixed independent of T and $p\geq -d\log T/\log\rho_{0},d>1,p=o\left({\left(\log T\right)}^{\bar{a}}\right)$ for some $\bar{a}<\infty$ the a.s. results of Lemma A.6 (I)-(III) and Lemma A.7 of [24] hold true for ${\left(\log T\right)}^{3}$ replaced by ${\left(\log T\right)}^{a}$ for some integer a<∞. In particular ${\bar{G}}_{p}-{\bar{\Gamma }}_{p}=O\left({\left(\log T\right)}^{a}/T^{1/2}\right)$.*

*(II) Using the notation $\delta G_{p}:={\bar{G}}_{p}-{\bar{\Gamma }}_{p}$ define*

$${\tilde{S}}_{T}:=\left[\begin{array}{cc} I_{c} & -\sqrt{T}\delta G_{•,1,p}^{\prime }\langle {\tilde{z}}_{t,•},{\tilde{z}}_{t,•}\rangle {\bar{\Gamma }}_{•,p}^{†}\\ 0 & I_{ps-c} \end{array}\right],\;{\bar{\Gamma }}_{•,p}^{†}:={\bar{\Gamma }}_{•,p}{\left({\bar{\Gamma }}_{•,p}^{\prime }\langle {\tilde{z}}_{t,•},{\tilde{z}}_{t,•}\rangle {\bar{\Gamma }}_{•,p}\right)}^{-1}.$$

*Then for ${\tilde{\Gamma }}_{p}^{\prime }:={\tilde{S}}_{T}{\tilde{D}}_{x}^{-1}{\bar{G}}_{p}^{\prime }{\tilde{D}}_{z}$ and*

$$\Gamma^{\prime }=\left[\begin{array}{cc} I & 0\\ 0 & \Gamma_{•}^{\prime } \end{array}\right]$$

*we obtain ${\tilde{\Gamma }}_{p}^{\prime }-{\left[\Gamma^{\prime }\right]}_{1:p}=\left[O\left({\left(\log T\right)}^{a}/T\right),O\left({\left(\log T\right)}^{a}/T^{1/2}\right)\right]$ where the partitioning corresponds to the partitioning of ${\tilde{z}}_{t,p}$ into ${\tilde{z}}_{t,1}$ and ${\tilde{z}}_{t,•}$. Here $\Gamma_{•}^{\prime }$ denotes the right factor of ${\tilde{b}}_{•,\infty }=O_{•}\Gamma_{•}^{\prime }$ such that ${\left[\Gamma_{•}^{\prime }\right]}_{1:p}S_{p,2}=I_{n-c}$ holds.*

*(III) Let the assumptions of Theorem 1 hold. Then ${\hat{Z}}_{T}:=Tvec\left(\left({\tilde{\Gamma }}_{p}^{\prime }-{\left[\Gamma^{\prime }\right]}_{1:p}\right)\left[\begin{array}{c} I_{c}\\ 0 \end{array}\right]\right)$ converges in distribution.*

**Proof.** (I) First consider the entries of the vectors ${\tilde{y}}_{t,•}$ and ${\tilde{z}}_{t,•}$ (see (A1)) more closely. Since in

$$O_{f,⊥}^{\prime }Y_{t,f}^{+}=O_{f,⊥}^{\prime }\left(O_{f,•}x_{t,•}+E_{f}E_{t,f}^{+}\right)$$

the nonstationary directions are filtered by definition, ${\tilde{y}}_{t,•}$ is stationary and does not depend on *T*. Further, also ${\tilde{z}}_{t,•}$ is stationary for fixed *p* as the nonstationary directions are either filtered by pre-multiplication with $O_{S,⊥}^{\prime }$ or by yearly differencing $Z_{t}-Z_{t-S}$. Therefore we obtain from stationary theory for fixed p=kS that

$$∥E{\tilde{y}}_{t,•}{\tilde{z}}_{t,•}^{\prime }{\left(E{\tilde{z}}_{t,•}{\tilde{z}}_{t,•}^{\prime }\right)}^{-1}-\langle {\tilde{y}}_{t,•},{\tilde{z}}_{t,•}\rangle {\langle {\tilde{z}}_{t,•},{\tilde{z}}_{t,•}\rangle }^{-1}∥=o\left(1\right).$$

Here $\sup_{p}∥{\left(E{\tilde{z}}_{t,•}{\tilde{z}}_{t,•}^{\prime }\right)}^{-1}∥<\infty$ has been discussed before. Now $E{\tilde{y}}_{t,•}{\tilde{z}}_{t,•}^{\prime }{\left(E{\tilde{z}}_{t,•}{\tilde{z}}_{t,•}^{\prime }\right)}^{-1}={\tilde{\beta }}_{•,p}+o\left(T^{-1/2}\right)=O_{•,p}{\left[\Gamma_{•}^{\prime }\right]}_{1:p}+o\left(T^{-1/2}\right)$ where the $o(T^{-1/2})$ term appears due to neglecting $\tilde{O_{f}}{\bar{A}}^{p}x_{t-p}$. It follows that $\det\left[{\left({\tilde{\beta }}_{•,p}S_{p,2}\right)}^{\prime }\left({\tilde{\beta }}_{•,p}S_{p,2}\right)\right]=\det\left[O_{•,p}^{\prime }O_{•,p}\right]>0$ and hence $∥{\hat{\beta }}_{•,p}-{\tilde{\beta }}_{•,p}{∥}_{Fr}=o\left(1\right)$ implies $\lim_{T\to \infty }\det\left[{\left({\hat{\beta }}_{•,p}S_{p,2}\right)}^{\prime }\left({\hat{\beta }}_{•,p}S_{p,2}\right)\right]>0$ a.s. where ${\hat{\beta }}_{•,p}:=\langle {\tilde{y}}_{t,•},{\tilde{z}}_{t,•}\rangle {\langle {\tilde{z}}_{t,•},{\tilde{z}}_{t,•}\rangle }^{-1}$. Since ${\hat{O}}_{•,p}{\bar{\Gamma }}_{•,p}^{\prime }-{\tilde{\beta }}_{•,p}=o\left(1\right)$ due to consistency, also

$$\lim_{T\to \infty }\det\left[{\left({\hat{O}}_{•,p}{\bar{\Gamma }}_{•,p}^{\prime }S_{p,2}\right)}^{\prime }\left({\hat{O}}_{•,p}{\bar{\Gamma }}_{•,p}^{\prime }S_{p,2}\right)\right]=\lim_{T\to \infty }\det{\hat{O}}_{•,p}^{\prime }{\hat{O}}_{•,p}\det{\left({\bar{\Gamma }}_{•,p}^{\prime }S_{p,2}\right)}^{2}>0\;a.s.$$

Since ${\bar{\Gamma }}_{•,p}-\Gamma_{•,p}=o\left(1\right)$ due to the definition of ${\bar{\Gamma }}_{•,p}$ and the continuity of the solution of the eigenvalue problem it follows that ${\hat{O}}_{•,p}-O_{•,p}=o\left(1\right)$ and therefore $\lim\sup_{T}\det{\hat{O}}_{•,p}^{\prime }{\hat{O}}_{•,p}>0$. As in Lemma 6 of [40] it can be shown that $\Gamma_{•,p}^{\prime }-{\left[\Gamma_{•}^{\prime }\right]}_{1:p}=o\left(T^{-1}\right)$ and $O_{•,p}=O_{•}+o\left(T^{-1}\right)$ for the range of *p* given in Theorem 1 since these matrices correspond to a stationary problem. Hence the chosen normalization of ${\bar{\Gamma }}_{•,p}$ can be used a.s. Next in order to obtain the convergence of $\bar{G}$ to ${\bar{\Gamma }}_{p}$, Lemma A.6 of BRRR is slightly extended to the current situation (for details and notation see there). Lemma A.6 of BRRR contains three parts: BRRR(I) gives bounds on the error in the matrices (with $l_{T}=\log T$)

$$\begin{array}{cc} \delta_{yz} & =\left[\begin{array}{cc} \frac{1}{T}\langle {\tilde{y}}_{t,1},{\tilde{z}}_{t,1}\rangle & \frac{1}{\sqrt{T}}\langle {\tilde{y}}_{t,1},{\tilde{z}}_{t,•}\rangle \\ \frac{1}{\sqrt{T}}\langle {\tilde{y}}_{t,•},{\tilde{z}}_{t,1}\rangle & \langle {\tilde{y}}_{t,•},{\tilde{z}}_{t,•}\rangle \end{array}\right]-\left[\begin{array}{cc} \frac{1}{T}\langle {\tilde{z}}_{t,1},{\tilde{z}}_{t,1}\rangle & 0\\ 0 & \langle {\tilde{y}}_{t,•},{\tilde{z}}_{t,•}\rangle \end{array}\right]=\left[\begin{array}{cc} O(\frac{1}{T}l_{T}^{a}) & O(\frac{1}{\sqrt{T}}l_{T}^{a})\\ O(\frac{1}{\sqrt{T}}l_{T}^{a}) & 0 \end{array}\right],\\ \delta_{yy} & =\left[\begin{array}{cc} \frac{1}{T}\langle {\tilde{y}}_{t,1},{\tilde{y}}_{t,1}\rangle & \frac{1}{\sqrt{T}}\langle {\tilde{y}}_{t,•},{\tilde{y}}_{t,•}\rangle \\ \frac{1}{\sqrt{T}}\langle {\tilde{y}}_{t,•},{\tilde{y}}_{t,1}\rangle & \langle {\tilde{y}}_{t,•},{\tilde{y}}_{t,•}\rangle \end{array}\right]-\left[\begin{array}{cc} \frac{1}{T}\langle {\tilde{z}}_{t,1},{\tilde{z}}_{t,1}\rangle & 0\\ 0 & \langle {\tilde{y}}_{t,•},{\tilde{y}}_{t,•}\rangle \end{array}\right]=\left[\begin{array}{cc} O(\frac{1}{T}l_{T}^{a}) & O(\frac{1}{\sqrt{T}}l_{T}^{a})\\ O(\frac{1}{\sqrt{T}}l_{T}^{a}) & 0 \end{array}\right],\\ \delta_{zz} & =\left[\begin{array}{cc} \frac{1}{T}\langle {\tilde{z}}_{t,1},{\tilde{z}}_{t,1}\rangle & \frac{1}{\sqrt{T}}\langle {\tilde{z}}_{t,1},{\tilde{z}}_{t,•}\rangle \\ \frac{1}{\sqrt{T}}\langle {\tilde{z}}_{t,•},{\tilde{z}}_{t,1}\rangle & \langle {\tilde{z}}_{t,•},{\tilde{z}}_{t,•}\rangle \end{array}\right]-\left[\begin{array}{cc} \frac{1}{T}\langle {\tilde{z}}_{t,1},{\tilde{z}}_{t,1}\rangle & 0\\ 0 & \langle {\tilde{z}}_{t,•},{\tilde{z}}_{t,•}\rangle \end{array}\right]=\left[\begin{array}{cc} 0 & O(\frac{1}{\sqrt{T}}l_{T}^{a})\\ O(\frac{1}{\sqrt{T}}l_{T}^{a}) & 0 \end{array}\right]. \end{array}$$

BRRR(II) deals with $J=\bar{Q}-\bar{\Phi }=$

$${\tilde{D}}_{z}\langle {\tilde{z}}_{t},{\tilde{y}}_{t}\rangle {\tilde{D}}_{y}{\left({\tilde{D}}_{y}\langle {\tilde{y}}_{t},{\tilde{y}}_{t}\rangle {\tilde{D}}_{y}\right)}^{-1}{\tilde{D}}_{y}\langle {\tilde{y}}_{t},{\tilde{z}}_{t}\rangle {\tilde{D}}_{z}-\left[\begin{array}{cc} \frac{1}{T}\langle {\tilde{z}}_{t,1},{\tilde{z}}_{t,1}\rangle & 0\\ 0 & \langle {\tilde{z}}_{t,•},{\tilde{y}}_{t,•}\rangle {\langle {\tilde{y}}_{t,•},{\tilde{y}}_{t,•}\rangle }^{-1}\langle {\tilde{y}}_{t,•},{\tilde{z}}_{t,•}\rangle \end{array}\right]$$

and BRRR(III) shows that there exists a solution ${\bar{G}}_{p}$ converging to a solution ${\bar{\Gamma }}_{p}$ of the separated problem. For showing the orders of convergence of $\delta_{zz}$ the arguments are unchanged except for noting that in $\langle {\tilde{z}}_{t,1},{\tilde{z}}_{t,•}\rangle$ the number of columns increases as a function of the sample size. Since the a.s. bounds on the entries of this expression hold uniformly (as follows straightforwardly from the arguments of Lemma A.1 of BRRR) this does not change the arguments. With respect to $\delta_{yz}$ note that now ${\tilde{y}}_{t}={\tilde{\beta }}_{1}{\tilde{z}}_{t,p}+{\tilde{\varepsilon }}_{t}+\tilde{O_{f}}{\bar{A}}^{p}x_{t-p}$. Due to the increase of *p* as a function of the sample size, ${\bar{A}}^{p}=o\left(T^{-1-\epsilon }\right)$ for small enough ϵ>0 and therefore $\tilde{O_{f}}{\bar{A}}^{p}x_{t-p}=o\left(T^{-1/2-\epsilon /2}\right)$ since $x_{t}=o\left(T^{(1+\epsilon )/2}\right)$ (uniformly in 1≤t≤T) whether ${\left(x_{t}\right)}_{t\in Z}$ is a unit root process or stationary. Hence $\langle \tilde{O_{f}}{\bar{A}}^{p}x_{t-p},\tilde{O_{f}}{\bar{A}}^{p}x_{t-p}\rangle =o\left(1\right)$. Further $\langle \tilde{O_{f}}{\bar{A}}^{p}x_{t-p},{\tilde{\varepsilon }}_{t}\rangle =o\left(T^{-1/2}\right)$ follows from $\langle x_{t-p},{\tilde{\varepsilon }}_{t}\rangle =O\left(\log T\right)$ (see Lemma A.1 (I)). This shows that the additional term is always of lower order and can be neglected. The remaining arguments follow exactly as in the proof of Lemma A.6 of BRRR. The proof of Lemma A.7 of BRRR only uses the order bounds derived above and hence follows immediately. This shows (I). (II) Using the definition of ${\tilde{S}}_{T}$ we obtain:

$$\begin{array}{ccc} {\tilde{\Gamma }}_{p}^{\prime } & = & {\tilde{S}}_{T}{\tilde{D}}_{x}^{-1}{\bar{G}}_{p}^{\prime }{\tilde{D}}_{z}={\tilde{S}}_{T}\left[\begin{array}{cc} I_{c} & \sqrt{T}\delta G_{•,1,p}^{\prime }\\ \delta G_{1,2,p}^{\prime }/\sqrt{T} & {\bar{G}}_{•,2,p}^{\prime } \end{array}\right]\\ & = & \left[\begin{array}{cc} I_{c}-\delta G_{•,1,p}^{\prime }\langle {\tilde{z}}_{t,•},{\tilde{z}}_{t,•}\rangle {\bar{\Gamma }}_{•,p}^{†}\delta G_{1,2,p}^{\prime } & \sqrt{T}\delta G_{•,1,p}^{\prime }\left(I-\langle {\tilde{z}}_{t,•},{\tilde{z}}_{t,•}\rangle {\bar{\Gamma }}_{•,p}^{†}{\bar{G}}_{•,2,p}^{\prime }\right)\\ \delta G_{1,2,p}^{\prime }/\sqrt{T} & {\bar{G}}_{•,2,p}^{\prime } \end{array}\right]. \end{array}$$

From (I) and Lemma A.7 of BRRR $\delta G_{•,1,p}=O\left({\left(\log T\right)}^{a}/T^{1/2}\right),\delta G_{1,2,p}=O\left({\left(\log T\right)}^{a}/T^{1/2}\right)$ and ${\bar{G}}_{•,2,p}-{\bar{\Gamma }}_{•,p}=o\left({\left(\log T\right)}^{a}/T^{1/2}\right)$. Finally

$$\delta G_{•,1,p}^{\prime }\left(I-\langle {\tilde{z}}_{t,•},{\tilde{z}}_{t,•}\rangle {\bar{\Gamma }}_{•,p}^{†}{\bar{G}}_{•,2,p}^{\prime }\right)=\delta G_{•,1,p}^{\prime }\left(I-\langle {\tilde{z}}_{t,•},{\tilde{z}}_{t,•}\rangle {\bar{\Gamma }}_{•,p}^{†}{\bar{\Gamma }}_{•,p}^{\prime }\right)+O\left({\left(\log T\right)}^{a}/T\right)=O\left({\left(\log T\right)}^{a}/T\right)$$

as in the proof of Lemma A.7 of BRRR. Using Lemma A.5 (III) of BRRR with ${\hat{\Xi }}_{f}={\langle {\tilde{y}}_{t,•},{\tilde{y}}_{t,•}\rangle }^{-1/2}$ it follows that ${\bar{\Gamma }}_{•,p}^{\prime }-\Gamma_{•,p}^{\prime }=O\left({\left(\log T\right)}^{a}T^{-1/2}\right)$. Since ${\bar{G}}_{•,2,p}-{\bar{\Gamma }}_{•,p}{=o(\left(\log T\right)}^{a}/$$T^{1/2})$ the same rate of convergence holds for ${\bar{G}}_{•,2,p}^{\prime }-\Gamma_{•,p}^{\prime }=O\left({\left(\log T\right)}^{a}/T^{1/2}\right)$. It follows that ${\tilde{\Gamma }}_{p}^{\prime }-{\left[\Gamma^{\prime }\right]}_{1:p}=\left[O\left({\left(\log T\right)}^{a}/T\right),O\left({\left(\log T\right)}^{a}/T^{1/2}\right)\right]$. (III) From above we have

(A3) $$T\left({\tilde{\Gamma }}_{p}^{\prime }-{\left[\Gamma^{\prime }\right]}_{1:p}\right)\left(\begin{array}{c} I_{c}\\ 0 \end{array}\right)=\left[\begin{array}{c} -(\sqrt{T}\delta G_{•,1,p}^{\prime }\langle {\tilde{z}}_{t,•},{\tilde{z}}_{t,•}\rangle {\bar{\Gamma }}_{•,p}^{†}\sqrt{T}\delta G_{1,2,p}^{\prime })\\ \sqrt{T}\delta G_{1,2,p}^{\prime } \end{array}\right]+o_{P}\left(1\right).$$

Now from the proof of Lemma A.7 of BRRR we obtain

$${\left[\sqrt{T}\delta G_{•,1,p}\right]}^{\prime }=\Xi O_{•}{\left(I-\Theta_{•}^{2}\right)}^{-1}{\bar{\Gamma }}_{•,p}^{\prime }+o_{P}\left(1\right).$$

Furthermore using the expression given in Lemma A.7 of BRRR:

$$\begin{array}{ccc} \sqrt{T}\delta G_{1,2,p} & = & \sqrt{T}Z_{11}^{-1}\left[\delta_{zz}^{1•}\Gamma_{•,p}\Theta_{•}^{2}-J_{1,•}\Gamma_{•,p}\right]{\left(I-\Theta_{•}^{2}\right)}^{-1}+o_{P}\left(1\right)\\ & = & \sqrt{T}Z_{11}^{-1}\left[\delta_{zz}^{1•}\Gamma_{•,p}\left(\Theta_{•}^{2}-I\right)+\left[\delta_{zz}^{1,•}-J_{1,•}\right]\Gamma_{•,p}\right]{\left(I-\Theta_{•}^{2}\right)}^{-1}+o_{P}\left(1\right)\\ & = & -Z_{11}^{-1}\langle {\tilde{z}}_{t,1},x_{t,•}\rangle -Z_{11}^{-1}\sqrt{T}\left[J_{1,•}-\delta_{zz}^{1,•}\right]\Gamma_{•,p}{\left(I-\Theta_{•}^{2}\right)}^{-1}+o_{P}\left(1\right)\\ & = & -Z_{11}^{-1}\langle {\tilde{z}}_{t,1},x_{t,•}\rangle -Z_{11}^{-1}E{\tilde{\varepsilon }}_{t,1}{\tilde{\varepsilon }}_{t,•}^{\prime }{\left(E{\tilde{y}}_{t,•}{\left({\tilde{y}}_{t,•}\right)}^{\prime }\right)}^{-1}E{\tilde{y}}_{t,•}x_{t,•}^{\prime }{\left(I-\Theta_{•}^{2}\right)}^{-1}+o_{P}\left(1\right). \end{array}$$

This shows the result.    □

The transformations in the representation lead to an estimator $\bar{G}$ taking the place of $\hat{K_{p}}$. Using ${\tilde{S}}_{T}$ as defined in Lemma A5 the corresponding estimator ${\tilde{\Gamma }}_{p}^{\prime }={\tilde{S}}_{T}{\tilde{D}}_{x}^{-1}{\bar{G}}_{p}^{\prime }{\tilde{D}}_{z}$ fulfills ${\tilde{\Gamma }}_{p}^{\prime }-\Gamma_{p}^{\prime }=\left[O\left({\left(\log T\right)}^{a}/T\right),O\left({\left(\log T\right)}^{a}/\sqrt{T}\right)\right]$.

Based on this result let (A,C,K) denote the realization of the true transfer function in the state basis corresponding to $\Gamma_{p}^{\prime }$ where $\Gamma_{p}^{\prime }S_{p}=I_{n}$ and let $\left(\tilde{A},\tilde{C},\tilde{K}\right)$ denote the (unfeasible) CVA estimates using ${\tilde{x}}_{t}:={\tilde{\Gamma }}_{p}^{\prime }{\tilde{z}}_{t,p}$. The next lemma then provides the main ingredients for the rest of the proofs:

**Lemma** **A6.**
*Let the assumptions of Theorem 1 hold and define $D_{x}:=diag\left(I_{c}T^{-1},I_{n-c}T^{-1/2}\right)$. Then there exists an integer a<∞ such that*

$$\begin{array}{ccc} \left(\tilde{A}-A\right)D_{x}^{-1}=O\left({\left(\log T\right)}^{a}\right), & \left(\tilde{C}-C\right)D_{x}^{-1}=O\left({\left(\log T\right)}^{a}\right), & \left(\tilde{K}-K\right)=O\left({\left(\log T\right)}^{a}/T^{1/2}\right). \end{array}$$

**Proof.** First note that the regression of $Y_{t,f}^{+}$ onto $Y_{t,p}^{-}$ includes time points t=p+1,...,T−f+1 whereas for estimating the system matrices we can use ${\hat{x}}_{t},t=p+1,...,T+1$ and $y_{t},t=p+1,...,T$. Thus in this proof we use ${\langle a_{t},b_{t}\rangle }_{p+1}^{T}:=T^{-1}\sum_{t=p+1}^{T}a_{t}b_{t}^{\prime }$ instead of $\langle a_{t},b_{t}\rangle =T^{-1}\sum_{t=p+1}^{T-f+1}a_{t}b_{t}^{\prime }$. The following orders of convergence are straightforward to derive using the results of Lemma A1, ${\bar{A}}^{p}=o\left(T^{-1}\right),\left({\tilde{\Gamma }}_{p}^{\prime }-{\left[\Gamma^{\prime }\right]}_{1:p}\right)D_{z}^{-1}=O\left({\left(\log T\right)}^{a}\right)$ and ${\tilde{x}}_{t}-x_{t}=\left({\tilde{\Gamma }}_{p}^{\prime }-{\left[\Gamma^{\prime }\right]}_{1:p}\right){\tilde{z}}_{t,p}-{\bar{A}}^{p}x_{t-p},t>p$ according to Lemma A5 and Lemma A1 for the range of *p* given in Theorem 1:

$$\begin{array}{ccc} {\langle \varepsilon_{t},{\tilde{x}}_{t}-x_{t}\rangle }_{p+1}^{T}=O\left(p{\left(\log T\right)}^{a}/T\right) & , & {\tilde{D}}_{z}{\langle {\tilde{z}}_{t,p},{\tilde{x}}_{t}-x_{t}\rangle }_{p+1}^{T}=O\left(p{\left(\log T\right)}^{a}/T^{1/2}\right)\\ {\tilde{D}}_{z}{\langle {\tilde{z}}_{t+1,p},{\tilde{x}}_{t}-x_{t}\rangle }_{p+1}^{T}=O\left(p{\left(\log T\right)}^{a}/T^{1/2}\right) & , & {\tilde{D}}_{x}{\langle x_{t},{\tilde{x}}_{t}-x_{t}\rangle }_{p+1}^{T}=O\left(p{\left(\log T\right)}^{a}/T^{1/2}\right)\\ {\langle {\tilde{x}}_{t}-x_{t},{\tilde{x}}_{t}-x_{t}\rangle }_{p+1}^{T}=O\left(p^{2}{\left(\log T\right)}^{a}/T\right) & . \end{array}$$

Using these orders of convergence we obtain

$${\tilde{D}}_{x}{\langle {\tilde{x}}_{t},{\tilde{x}}_{t}\rangle }_{p+1}^{T}{\tilde{D}}_{x}={\tilde{D}}_{x}{\langle x_{t},x_{t}\rangle }_{p+1}^{T}{\tilde{D}}_{x}+O\left(p^{2}{\left(\log T\right)}^{a}/T^{1/2}\right)>0\;a.s.$$

From Lemma A1 also ${\left({\tilde{D}}_{x}{\langle {\tilde{x}}_{t},{\tilde{x}}_{t}\rangle }_{p+1}^{T}{\tilde{D}}_{x}\right)}^{-1}={\left({\tilde{D}}_{x}{\langle x_{t},x_{t}\rangle }_{p+1}^{T}{\tilde{D}}_{x}\right)}^{-1}\left(1+o\left(1\right)\right)=O\left({\left(\log T\right)}^{a}\right)$. Therefore

(A4) $$\begin{array}{ccc} \left(\tilde{C}-C\right)D_{x}^{-1} & = & \sqrt{T}\left({\langle \varepsilon_{t},{\tilde{x}}_{t}\rangle }_{p+1}^{T}-{C\langle {\tilde{x}}_{t}-x_{t},{\tilde{x}}_{t}\rangle }_{p+1}^{T}\right){\tilde{D}}_{x}{\left({\tilde{D}}_{x}{\langle {\tilde{x}}_{t},{\tilde{x}}_{t}\rangle }_{p+1}^{T}{\tilde{D}}_{x}\right)}^{-1}\\ & = & \sqrt{T}{\langle \varepsilon_{t},x_{t}\rangle }_{p+1}^{T}{\tilde{D}}_{x}{\left({\tilde{D}}_{x}{\langle x_{t},x_{t}\rangle }_{p+1}^{T}{\tilde{D}}_{x}+o\left(1\right)\right)}^{-1}\\ & & -\sqrt{T}C{\langle {\tilde{x}}_{t}-x_{t},x_{t}\rangle }_{p+1}^{T}{\tilde{D}}_{x}{\left({\tilde{D}}_{x}{\langle x_{t},x_{t}\rangle }_{p+1}^{T}{\tilde{D}}_{x}\right)}^{-1}+o\left(1\right)=O\left(p{\left(\log T\right)}^{a}\right). \end{array}$$

This in particular establishes consistency for the estimate. Next analogously (using the notation $\delta x_{t}={\tilde{x}}_{t}-x_{t}$) we obtain $\left(\tilde{A}-A\right)D_{x}^{-1}=$

(A5) $$\begin{array}{ccc} & & \sqrt{T}{\langle {\tilde{x}}_{t+1}-A{\tilde{x}}_{t},{\tilde{x}}_{t}\rangle }_{p+1}^{T}{\tilde{D}}_{x}{\left({\tilde{D}}_{x}{\langle {\tilde{x}}_{t},{\tilde{x}}_{t}\rangle }_{p+1}^{T}{\tilde{D}}_{x}\right)}^{-1}\\ & = & \sqrt{T}\left({\langle \left({\tilde{x}}_{t+1}-x_{t+1}\right)+\left(x_{t+1}-Ax_{t}\right)+A\left(x_{t}-{\tilde{x}}_{t}\right),{\tilde{x}}_{t}\rangle }_{p+1}^{T}{\tilde{D}}_{x}\right){\left({\tilde{D}}_{x}{\langle x_{t},x_{t}\rangle }_{p+1}^{T}{\tilde{D}}_{x}+o\left(1\right)\right)}^{-1}\\ & = & \sqrt{T}\left({\langle \delta x_{t+1},x_{t}\rangle }_{p+1}^{T}-A{\langle \delta x_{t},x_{t}\rangle }_{p+1}^{T}+{\langle K\varepsilon_{t},x_{t}\rangle }_{p+1}^{T}\right){\tilde{D}}_{x}{\left({\tilde{D}}_{x}{\langle x_{t},x_{t}\rangle }_{p+1}^{T}{\tilde{D}}_{x}\right)}^{-1}+o\left(1\right)\\ & = & O(p{\left(\log T\right)}^{a}) \end{array}$$

and therefore consistency for $\tilde{A}$ is established. Finally note that for

$${\hat{\varepsilon }}_{t}=y_{t}-\tilde{C}{\tilde{x}}_{t}=\varepsilon_{t}+C(x_{t}-{\tilde{x}}_{t})+\left(C-\tilde{C}\right){\tilde{x}}_{t}$$

it follows that ${\langle {\hat{\varepsilon }}_{t},{\hat{\varepsilon }}_{t}\rangle }_{p+1}^{T}=\Omega +O\left(p^{2}{\left(\log T\right)}^{a}/T^{1/2}\right)$. Furthermore since ${\hat{\varepsilon }}_{t}$ denotes the residuals of the regression of $y_{t}$ onto ${\tilde{x}}_{t}$ it follows that ${\langle {\hat{\varepsilon }}_{t},{\bar{x}}_{t}\rangle }_{p+1}^{T}=0$. From this we obtain

(A6) $$\begin{array}{ccc} \sqrt{T}\left(\tilde{K}-K\right) & = & \sqrt{T}({\langle {\tilde{x}}_{t+1}-K{\hat{\varepsilon }}_{t},{\hat{\varepsilon }}_{t}\rangle }_{p+1}^{T}{\left({\langle {\hat{\varepsilon }}_{t},{\hat{\varepsilon }}_{t}\rangle }_{p+1}^{T}\right)}^{-1}\\ & = & \sqrt{T}\left({\langle \left({\tilde{x}}_{t+1}-x_{t+1}\right)-A\delta x_{t}+K\left(\varepsilon_{t}-{\hat{\varepsilon }}_{t}\right),{\hat{\varepsilon }}_{t}\rangle }_{p+1}^{T}\right){\left({\langle {\hat{\varepsilon }}_{t},{\hat{\varepsilon }}_{t}\rangle }_{p+1}^{T}\right)}^{-1}\\ & = & \sqrt{T}\left({\langle \delta x_{t+1}-A\delta x_{t}+K\left(\varepsilon_{t}-{\hat{\varepsilon }}_{t}\right),{\hat{\varepsilon }}_{t}\rangle }_{p+1}^{T}\right)\Omega^{-1}\left(1+o\left(1\right)\right)\\ & = & \sqrt{T}\left({\langle \delta x_{t+1}-A\delta x_{t}+K\left(\varepsilon_{t}-{\hat{\varepsilon }}_{t}\right),\varepsilon_{t}\rangle }_{p+1}^{T}\right)\Omega^{-1}\left(1+o\left(1\right)\right)+o\left(1\right)\\ & = & \left(\sqrt{T}{\langle \delta x_{t+1},\varepsilon_{t}\rangle }_{p+1}^{T}\right)\Omega^{-1}+o\left(1\right)=\left(\sqrt{T}{\langle \left({\tilde{\Gamma }}_{p}^{\prime }-\Gamma_{p}^{\prime }\right){\bar{z}}_{t+1,p},\varepsilon_{t}\rangle }_{p+1}^{T}\right)\Omega^{-1}+o\left(1\right)\\ & = & O(p{\left(\log T\right)}^{a}). \end{array}$$

   □

These expressions do not only show consistency of a specific order, but also give the relevant highest order terms for the asymptotic distribution, which are used below.

As $\hat{C}{\hat{A}}^{j}\hat{K}=\tilde{C}{\tilde{A}}^{j}\tilde{K}\to CA^{j}K=C_{\circ }A_{\circ }^{j}K_{\circ }$, Lemma A6 establishes consistency for the impulse response sequence $\hat{C}{\hat{A}}^{j}\hat{K}$ (thus proofs Theorem 1 (I)) as well as, jointly with $p=O({\left(\log T\right)}^{a})$, the rate of convergence $O({\left(\log T\right)}^{a}/T^{1/2})$ for the not realizable choice of the basis and the impulse response sequence $CA^{j}K$.

#### Appendix C.1.2. Proof of Theorem 1 (II)

In order to arrive at the canonical representation $\left(\overset{ˇ}{A},\overset{ˇ}{C},\overset{ˇ}{K}\right)$ two steps are performed: first the reordered Jordan normal form is calculated, afterwards the matrices ${\tilde{C}}_{j,C}$ are transformed such that $E_{j}^{\prime }{\overset{ˇ}{C}}_{j,C}=I_{c_{j}}$ holds. We will follow these steps below.

In the first step a transformation matrix $\hat{U}$ needs to be found such that $\tilde{A}=\hat{U}\tilde{A}{\hat{U}}^{-1}$ is in reordered Jordan normal form. In this respect $\tilde{A}$ and A are used in Lemma A2. Accordingly ${\hat{U}}_{t}=\left[{\hat{U}}_{t,1},...,{\hat{U}}_{t,S/2},{\hat{U}}_{t,•}\right]$ can be defined such that $V_{j}^{\prime }{\hat{U}}_{t,j}=I_{c_{j}}$ where $U\in R^{n\times n}$ corresponds to the transformation from A to $A_{\circ }$ as given in the theorem. An appropriate choice of ${\tilde{z}}_{t,1}$ leads to $U=I_{n}$. Furthermore the basis in the space spanned by the columns of ${\hat{U}}_{t,•}$ where ${\hat{U}}_{t,j}^{\prime }{\hat{U}}_{t,•}=0$ can be chosen such that $\left[0,I\right]{\hat{U}}_{t,•}=I$ for large enough *T*.

A first order approximation according to Lemma A2 then leads to

$${\hat{U}}_{t,j}=U_{j}+Z_{1}+O(∥\hat{A}{-A∥}^{2})=U_{j}-\Sigma \left(\hat{A}-A\right)U_{j}+O(∥\hat{A}-A{∥}^{2})$$

for j=0,...,S/2. Consequently $∥{\hat{U}}_{t,j}-U_{j}∥=O\left({\left(\log T\right)}^{a}T^{-1}\right)$ and thus also ${\hat{U}}_{t}-U=O\left({\left(\log T\right)}^{a}T^{-1}\right)$. Consequently the order of convergence for the transformed system $\left(\hat{A},\hat{C},\hat{K}\right)$ is unchanged. In a second step an upper triangular transformation matrix $\tilde{U}$ can be found transforming $\left(\hat{A},\hat{C},\hat{K}\right)$ such that $\tilde{A}$ corresponds to the reordered Jordan normal form. Due to the upper block triangularity of this transform we can apply Lemma A3 to show that the order of convergence remains identical.

For the second step note that Lemma A3 provides the required terms: An application to the block diagonal transformation $S_{T}=\mathrm{diag}\left(E_{1}^{\prime }{\tilde{C}}_{1,C},...,E_{S/2}^{\prime }{\tilde{C}}_{S/2,C},S_{T,•}\right)$, where $S_{T,•}$ transforms the stationary subsystem to echelon form, concludes the proof.

#### Appendix C.1.3. Proof of Theorem 1 (III)

The only argument that uses the iid assumption is the almost sure convergence of ${\left({\tilde{D}}_{x}\langle x_{t},x_{t}\rangle {\tilde{D}}_{x}\right)}^{-1}$. Weakening the assumptions on the noise implies that this order of convergence still holds in probability while the almost sure version cannot be shown with the tools of this paper. This concludes the proof of Theorem 1.

### Appendix C.2. Proof of Theorem 2

Using the notation introduced in (A1),

$$\hat{X}={\tilde{D}}_{z}\langle {\tilde{y}}_{t},{\tilde{z}}_{t,p}\rangle {\tilde{D}}_{z}{\left({\tilde{D}}_{z}\langle {\tilde{z}}_{t,p},{\tilde{z}}_{t,p}\rangle {\tilde{D}}_{z}\right)}^{-1}{\tilde{D}}_{z}\langle {\tilde{z}}_{t,p},{\tilde{y}}_{t}\rangle {\tilde{D}}_{z}{\left({\tilde{D}}_{z}\langle {\tilde{y}}_{t},{\tilde{y}}_{t}\rangle {\tilde{D}}_{z}\right)}^{-1}\to X_{\circ }=\left[\begin{array}{cc} I_{c} & 0\\ 0 & X_{\circ ,•} \end{array}\right]$$

for a suitable matrix $X_{\circ ,•}$. The eigenvalues of $\hat{X}$ are the squares of the singular values of the RRR problem in the first step of CVA. Therefore

$$\begin{array}{ccc} T\sum_{i=1}^{c}\left(1-{\hat{\sigma }}_{i}^{2}\right) & = & -Ttr\left[U_{c}^{\prime }\left(\hat{X}-X_{\circ }\right)\left[U_{c}-{\left(X_{\circ }-I\right)}^{†}\left(\hat{X}-X_{\circ }\right)U_{c}\right]\right]+o_{P}\left(1\right)\\ & = & -Ttr\left[\Delta X_{11}-\Delta X_{12}{\left(X_{\circ ,•}-I\right)}^{†}\Delta X_{21}\right]+o_{P}\left(1\right) \end{array}$$

according to a second order approximation in the Rayleigh-Schrödinger expansions (Lemma A2). Now, in the current situation we obtain $\left(I-\hat{X}\right)\left[\begin{array}{c} I\\ 0 \end{array}\right]=$

$$\begin{array}{ccc} & = & \left({\tilde{D}}_{y}\langle {\tilde{y}}_{t},{\tilde{y}}_{t}\rangle {\tilde{D}}_{y}-{\tilde{D}}_{y}\langle {\tilde{y}}_{t},{\tilde{z}}_{t,p}\rangle {\tilde{D}}_{z}{\left({\tilde{D}}_{z}\langle {\tilde{z}}_{t,p},{\tilde{z}}_{t,p}\rangle {\tilde{D}}_{z}\right)}^{-1}{\tilde{D}}_{z}\langle {\tilde{z}}_{t,p},{\tilde{y}}_{t}\rangle {\tilde{D}}_{y}\right){\left({\tilde{D}}_{y}\langle {\tilde{y}}_{t},{\tilde{y}}_{t}\rangle {\tilde{D}}_{y}\right)}^{-1}\left[\begin{array}{c} I\\ 0 \end{array}\right]\\ & = & \left({\tilde{D}}_{y}\langle {\tilde{\varepsilon }}_{t},{\tilde{\varepsilon }}_{t}\rangle {\tilde{D}}_{y}-{\tilde{D}}_{y}\langle {\tilde{\varepsilon }}_{t},{\tilde{z}}_{t,p}\rangle {\tilde{D}}_{z}{\left({\tilde{D}}_{z}\langle {\tilde{z}}_{t,p},{\tilde{z}}_{t,p}\rangle {\tilde{D}}_{z}\right)}^{-1}{\tilde{D}}_{z}\langle {\tilde{z}}_{t,p},{\tilde{\varepsilon }}_{t}\rangle {\tilde{D}}_{y}\right){\left({\tilde{D}}_{y}\langle {\tilde{y}}_{t},{\tilde{y}}_{t}\rangle {\tilde{D}}_{y}\right)}^{-1}\left[\begin{array}{c} I\\ 0 \end{array}\right]. \end{array}$$

Furthermore $\langle {\tilde{\varepsilon }}_{t},{\tilde{z}}_{t,p}\rangle {\tilde{D}}_{z}{\left({\tilde{D}}_{z}\langle {\tilde{z}}_{t,p},{\tilde{z}}_{t,p}\rangle {\tilde{D}}_{z}\right)}^{-1}{\tilde{D}}_{z}\langle {\tilde{z}}_{t,p},{\tilde{\varepsilon }}_{t}\rangle =O_{P}\left(T^{-1}\right)$ and

$${\left({\tilde{D}}_{y}\langle {\tilde{y}}_{t},{\tilde{y}}_{t}\rangle {\tilde{D}}_{y}\right)}^{-1}\left[\begin{array}{c} I\\ 0 \end{array}\right]=\left[\begin{array}{c} I\\ -{\langle {\tilde{y}}_{t,•},{\tilde{y}}_{t,•}\rangle }^{-1}\langle {\tilde{y}}_{t,•},{\tilde{y}}_{t,1}\rangle /\sqrt{T} \end{array}\right]{\left(\langle {\tilde{y}}_{t,1}^{\pi },{\tilde{y}}_{t,1}^{\pi }\rangle /T\right)}^{-1}$$

where ${\tilde{y}}_{t,1}^{\pi }={\tilde{y}}_{t,1}-\langle {\tilde{y}}_{t,1},{\tilde{y}}_{t,•}\rangle {\langle {\tilde{y}}_{t,•},{\tilde{y}}_{t,•}\rangle }^{-1}{\tilde{y}}_{t,•}.$

From this we get using $E{\tilde{\varepsilon }}_{t,•}{\tilde{\varepsilon }}_{t,•}^{\prime }=E{\tilde{y}}_{t,•}{\tilde{y}}_{t,•}^{\prime }-X_{\circ ,•}E{\tilde{y}}_{t,•}{\tilde{y}}_{t,•}^{\prime }$:

$$\begin{array}{ccc} T(I_{c}-{\hat{X}}_{1,1}) & = & \left(\langle {\tilde{\varepsilon }}_{t,1},{\tilde{\varepsilon }}_{t,1}\rangle -\langle {\tilde{\varepsilon }}_{t,1},{\tilde{\varepsilon }}_{t,•}\rangle {\langle {\tilde{y}}_{t,•},{\tilde{y}}_{t,•}\rangle }^{-1}\langle {\tilde{y}}_{t,•},{\tilde{y}}_{t,1}\rangle \right){\left(\langle {\tilde{y}}_{t,1}^{\pi },{\tilde{y}}_{t,1}^{\pi }\rangle /T\right)}^{-1}+o_{P}\left(1\right),\\ \sqrt{T}\Delta X_{2,1} & = & \left(-\langle {\tilde{\varepsilon }}_{t,•},{\tilde{\varepsilon }}_{t,1}\rangle +\left(I-X_{\circ ,•}\right)\langle {\tilde{y}}_{t,•},{\tilde{y}}_{t,1}\rangle \right){\left(\langle {\tilde{y}}_{t,1}^{\pi },{\tilde{y}}_{t,1}^{\pi }\rangle /T\right)}^{-1}+o_{P}\left(1\right)\\ \sqrt{T}\Delta X_{1,2} & = & -E{\tilde{\varepsilon }}_{t,1}{\tilde{\varepsilon }}_{t,•}^{\prime }{\left(E{\tilde{y}}_{t,•}{\tilde{y}}_{t,•}^{\prime }\right)}^{-1}+o_{P}\left(1\right). \end{array}$$

Thus $T\sum_{i=1}^{c}\left(1-{\hat{\sigma }}_{i}^{2}\right)=$

$$\begin{array}{ccc} & = & tr\left[\left(\langle {\tilde{\varepsilon }}_{t,1},{\tilde{\varepsilon }}_{t,1}\rangle -E{\tilde{\varepsilon }}_{t,1}{\tilde{\varepsilon }}_{t,•}^{\prime }{\left(E{\tilde{y}}_{t,•}{\tilde{y}}_{t,•}^{\prime }\right)}^{-1}{\left(I-X_{0,•}\right)}^{-1}E{\tilde{\varepsilon }}_{t,•}{\tilde{\varepsilon }}_{t,1}^{\prime }\right){\left(\langle {\tilde{y}}_{t,1},{\tilde{y}}_{t,1}\rangle /T\right)}^{-1}\right]+o_{P}\left(1\right)\\ & = & tr\left[\left(\langle {\tilde{\varepsilon }}_{t,1},{\tilde{\varepsilon }}_{t,1}\rangle -E{\tilde{\varepsilon }}_{t,1}{\tilde{\varepsilon }}_{t,•}^{\prime }{\left(E{\tilde{\varepsilon }}_{t,•}{\tilde{\varepsilon }}_{t,•}^{\prime }\right)}^{-1}E{\tilde{\varepsilon }}_{t,•}{\tilde{\varepsilon }}_{t,1}^{\prime }\right){\left(\langle {\tilde{y}}_{t,1},{\tilde{y}}_{t,1}\rangle /T\right)}^{-1}\right]+o_{P}\left(1\right)\overset{d}{\to }Z. \end{array}$$

### Appendix C.3. Proof of Theorem 3

The proof of Theorem 3 follows the same path as the proof of Theorem 1. In (A5) it was shown that the asymptotic distribution of $T({\tilde{A}}_{11}-A_{\circ ,11})$ depends on

$$\begin{array}{ccc} \langle {\tilde{x}}_{t+1,j}-x_{t+1,j},x_{t,k}\rangle ,\langle {\tilde{x}}_{t,j}-x_{t,j},x_{t,k}\rangle & , & \langle \varepsilon_{t},x_{t,j}\rangle ,\langle x_{t,k},x_{t,j}\rangle /T \end{array}$$

for j,k=0,...,S/2. Note that

$$\delta x_{t+i}={\tilde{x}}_{t+i}-x_{t+i}=\left({\tilde{\Gamma }}_{p}^{\prime }-{\left[\Gamma^{\prime }\right]}_{1:p}\right){\tilde{z}}_{t+i,p}+o_{P}\left(T^{-1}\right)$$

for i=0,1. Then the results of Lemma A5 show that the first *c* columns of $\left({\tilde{\Gamma }}_{p}^{\prime }-{\left[\Gamma^{\prime }\right]}_{1:p}\right)$ converge to a random variable (below denoted as $Z_{\Gamma }$) when multiplied with *T* while the remaining columns converge in distribution when multiplied with $\sqrt{T}$. Therefore

$$\langle \delta x_{t+i},x_{t,k}\rangle =T\left({\tilde{\Gamma }}_{p}^{\prime }-{\left[\Gamma^{\prime }\right]}_{1:p}\right)\frac{\langle {\tilde{z}}_{t+i,p},x_{t,k}\rangle }{T}+o_{P}\left(1\right)=T\left({\tilde{\Gamma }}_{p}^{\prime }-{\left[\Gamma^{\prime }\right]}_{1:p}\right)\left[\begin{array}{c} I_{c}\\ 0 \end{array}\right]\frac{\langle {\tilde{z}}_{t+i,1},x_{t,k}\rangle }{T}+o_{P}\left(1\right).$$

Due to the definition (A1), ${\tilde{z}}_{t,1}={\left[x_{t,j}\right]}_{j=0,...,S/2}+o\left(T^{-1}\right)$ and hence (using $A_{\circ }=diag\left(A_{\circ ,u},A_{\circ ,•}\right)$)

$$\langle {\tilde{z}}_{t+1,1},x_{t,k}\rangle /T=A_{\circ ,u}\langle {\tilde{z}}_{t,1},x_{t,k}\rangle /T+o\left(1\right).$$

Considering now the complex-valued representation and using the notation

$$\Delta \Gamma_{1}:=T\left({\tilde{\Gamma }}_{p}^{\prime }-{\left[\Gamma^{\prime }\right]}_{1:p}\right)\left[\begin{array}{c} I_{c}\\ 0 \end{array}\right],\;S_{j}=\left[0_{c_{j},\sum_{i<j}c_{i}},I_{c_{j}},0_{c_{j},\sum_{i>j}c_{j}}\right]$$

where $S_{j}{\tilde{z}}_{t,1}=x_{t,j,C}$, it follows that the contribution of these two terms to the limiting distribution of the diagonal block corresponding to the unit root $z_{j}$ amounts to (using $\langle x_{t,j,C},x_{t,k,C}\rangle /T\to 0$ for k≠j and $\delta x_{t,j,C}={\tilde{x}}_{t,j,C}-x_{t,j,C}$)

$$\langle \delta x_{t+1,j,C},x_{t,j,C}\rangle -A_{\circ ,jj}\langle \delta x_{t,j,C},x_{t,j,C}\rangle =$$

$$\begin{array}{ccc} & = & S_{j}\Delta \Gamma_{1}A_{\circ ,u}\frac{\langle {\tilde{z}}_{t,1},x_{t,j,C}\rangle }{T}-A_{\circ ,jj}S_{j}\Delta \Gamma_{1}\frac{\langle {\tilde{z}}_{t,1},x_{t,j,C}\rangle }{T}+o_{P}\left(1\right)\\ & = & S_{j}\Delta \Gamma_{1}S_{j}^{\prime }A_{\circ ,jj}\frac{\langle x_{t,j,C},x_{t,j,C}\rangle }{T}-A_{\circ ,jj}S_{j}\Delta \Gamma_{1}S_{j}^{\prime }\frac{\langle x_{t,j,C},x_{t,j,C}\rangle }{T}+o_{P}\left(1\right)\\ & = & S_{j}\Delta \Gamma_{1}S_{j}^{\prime }\bar{z_{j}}\frac{\langle x_{t,j,C},x_{t,j,C}\rangle }{T}-\bar{z_{j}}S_{j}\Delta \Gamma_{1}S_{j}^{\prime }\frac{\langle x_{t,j,C},x_{t,j,C}\rangle }{T}+o_{P}\left(1\right)=o_{P}\left(1\right). \end{array}$$

Therefore, for the diagonal blocks in (A5) these two terms do not contribute and the asymptotic distribution is determined by

$$T\langle K_{\circ ,j}\varepsilon_{t},x_{t,j}\rangle {\langle x_{t,j},x_{t,j}\rangle }^{-1}$$

for which the asymptotic results are provided in Lemma A1. This also shows that estimating the state does not change the asymptotic distribution in the diagonal blocks as the impact of ${\tilde{\Gamma }}_{p}-\Gamma_{p}$ is of lower order.

In order to derive the distribution of the sum of the eigenvalues note that as in the proof of Theorem 2, according to Lemma A2 the sum of the eigenvalues of $\tilde{A}$ converging to $z_{j}$ obeys the following second order approximation:

$$\begin{array}{ccc} T\sum_{i=1}^{c_{j}}\left({\hat{\lambda }}_{i}-z_{j}\right) & = & Ttr\left[U_{j}^{\prime }\left(\hat{A}-A_{\circ }\right)\left[U_{j}-A_{\circ }{\left(z_{j}\right)}^{†}\left(\hat{A}-A_{\circ }\right)U_{j}\right]\right]+o_{P}\left(T^{-1}\right)\\ & = & Ttr\left[{\hat{A}}_{\circ ,jj}-z_{j}I_{c_{j}}\right]+o_{P}\left(1\right) \end{array}$$

since $\left(\hat{A}-A_{\circ }\right)U_{j}=O\left({\left(\log T\right)}^{a}T^{-1}\right)$ in this case implying that the second order terms vanish. Thus we obtain the asymptotic distribution under the null hypothesis as the limiting distribution of

$$Ttr[\langle K_{\circ ,j,C}\varepsilon_{t},x_{t,j,C}\rangle {\langle x_{t,j,C},x_{t,j,C}\rangle }^{-1}].$$

It is easy to verify that this test statistic is pivotal for complex and real unit roots. This proves Theorem 3.

### Appendix C.4. Proof of Theorem 4

The result for ${\tilde{C}}_{m}$ can be shown using the results of [4]. As the eigenvalues are insensitive to changes in the basis we can assume without restriction of generality that the only unit root components in $TX_{t}^{\left(m\right)}$ are contained in the first $c_{m}$ rows:

$$c_{t}^{\left(m\right)}:=TX_{t}^{\left(m\right)}=\left[\begin{array}{c} c_{t,u}^{\left(m\right)}\\ c_{t,•}^{\left(m\right)} \end{array}\right],\;{\tilde{D}}_{c}=\left[\begin{array}{cc} T^{-1}I_{c_{m}} & 0\\ 0 & I_{n-c_{m}} \end{array}\right].$$

Due to the filtering, $c_{t,•}^{\left(m\right)}$ is stationary while $c_{t,u}^{\left(m\right)}$ contains the unit root $z_{m}$. Then the relevant matrix ${\hat{X}}_{m}$ can be written as

$${\hat{X}}_{m}:=\langle c_{t-1}^{\pi },p_{t}^{\pi }\rangle {\langle p_{t}^{\pi },p_{t}^{\pi }\rangle }^{-1}\langle p_{t}^{\pi },c_{t-1}^{\pi }\rangle {\langle c_{t-1}^{\pi },c_{t-1}^{\pi }\rangle }^{-1}.$$

Since $p_{t}=K\varepsilon_{t-1}+\sum_{j=1,j\neq m}^{S}\alpha_{j}\beta_{j}^{\prime }X_{t-1}^{\left(j\right)}+\left[0,{\tilde{\alpha }}_{m}\right]c_{t-1}^{\left(m\right)},$ we consequently have $p_{t}^{\pi }=K\varepsilon_{t-1}^{\pi }+\left[0,{\tilde{\alpha }}_{m}\right]c_{t-1}^{\pi }$. Therefore, for the three components of ${\hat{X}}_{m}$ we obtain with appropriate definitions of the random variables $S_{m},T_{m}$ and using standard asymptotics

$$\begin{array}{ccc} \langle p_{t}^{\pi },p_{t}^{\pi }\rangle =\langle K\varepsilon_{t-1}^{\pi }+{\tilde{\alpha }}_{m}c_{t-1,•}^{\pi },K\varepsilon_{t-1}^{\pi }+{\tilde{\alpha }}_{m}c_{t-1,•}^{\pi }\rangle & \to & K\left(E\varepsilon_{t-1}\varepsilon_{t-1}^{\prime }\right)K^{\prime }+{\tilde{\alpha }}_{m}Ec_{t-1,•}^{\Pi }{\left(c_{t-1,•}^{\Pi }\right)}^{\prime }{\tilde{\alpha }}_{m}^{\prime }>0,\\ \langle p_{t}^{\pi },c_{t-1}^{\pi }\rangle =\langle K\varepsilon_{t-1}^{\pi }+{\tilde{\alpha }}_{m}c_{t-1,•}^{\pi },c_{t-1}^{\pi }\rangle & \overset{d}{\to } & [S_{m},{\tilde{\alpha }}_{m}Ec_{t-1,•}^{\Pi }{\left(c_{t-1,•}^{\Pi }\right)}^{\prime }],\\ \langle p_{t}^{\pi },c_{t-1}^{\pi }\rangle {\langle c_{t-1}^{\pi },c_{t-1}^{\pi }\rangle }^{-1}{\tilde{D}}_{c}^{-1} & = & \left[0,{\tilde{\alpha }}_{m}\right]+\langle K\varepsilon_{t-1}^{\pi },c_{t-1}^{\pi }\rangle {\langle c_{t-1}^{\pi },c_{t-1}^{\pi }\rangle }^{-1}{\tilde{D}}_{c}^{-1}\\ \langle K\varepsilon_{t-1}^{\pi },c_{t-1}^{\pi }\rangle {\langle c_{t-1}^{\pi },c_{t-1}^{\pi }\rangle }^{-1}{\tilde{D}}_{c}^{-1} & \overset{d}{\to } & [T_{m},0]. \end{array}$$

Correspondingly the first block column ${\hat{X}}_{m,u}$ of ${\hat{X}}_{m}$ converges to zero such that $T{\hat{X}}_{m,u}$ converges in distribution while the second block column converges in probability without normalization. This shows that

$$\begin{array}{ccc} T\sum_{i=1}^{c_{m}}{\hat{\lambda }}_{i} & = & Ttr\left[U_{m}^{\prime }\left({\hat{X}}_{m}-X_{m}\right)[U_{m}-X_{m}^{†}\left({\hat{X}}_{m}-X_{m}\right)U_{m}\right]+o_{P}\left(1\right)=tr\left[T{\hat{X}}_{m,uu}\right]+o_{P}\left(1\right) \end{array}$$

converges in distribution. The limit is given in [4].

For the case of the estimated state note that the difference between the estimated and the true state is given as

$${\tilde{x}}_{t}-x_{t}={\tilde{\Gamma }}_{p}^{\prime }{\tilde{z}}_{t,p}-\Gamma_{p}^{\prime }{\tilde{z}}_{t,p}-{\bar{A}}^{p}x_{t-p}={\left({\tilde{\Gamma }}_{p}-\Gamma_{p}\right)}^{\prime }{\tilde{z}}_{t,p}-{\bar{A}}^{p}x_{t-p}.$$

The strict minimum-phase assumption and the assumption on the increase of p=p(T) implies that the second term can be neglected being $o_{P}\left(T^{-1}\right)$. Furthermore

$${\left({\tilde{\Gamma }}_{p}-\Gamma_{p}\right)}^{\prime }{\tilde{z}}_{t,p}={\left({\tilde{\Gamma }}_{p}-\Gamma_{p}\right)}^{\prime }{\tilde{D}}_{z}^{-1}{\tilde{D}}_{z}{\tilde{z}}_{t,p},\;{\left({\tilde{\Gamma }}_{p}-\Gamma_{p}\right)}^{\prime }{\tilde{D}}_{z}^{-1}=O_{P}\left(T^{-1/2}\right).$$

Using this it can be concluded that

$$\begin{array}{ccc} \langle {\hat{p}}_{t},{\hat{p}}_{t}\rangle =\langle p_{t},p_{t}\rangle +O_{P}\left(T^{-1/2}\right) & , & \langle {\hat{p}}_{t},{\hat{c}}_{t-1,•}^{\left(m\right)}\rangle =\langle p_{t},c_{t-1,•}^{\left(m\right)}\rangle +O_{P}\left(T^{-1/2}\right),\\ \langle {\hat{p}}_{t},{\hat{c}}_{t-1,u}^{\left(m\right)}\rangle =\langle p_{t},c_{t-1,u}^{\left(m\right)}\rangle +O_{P}\left(T^{-1/2}\right) & , & \langle {\hat{c}}_{t,u}^{\left(m\right)},{\hat{c}}_{t,u}^{\left(k\right)}\rangle =\langle c_{t,u}^{\left(m\right)},c_{t,u}^{\left(k\right)}\rangle +O_{P}\left(1\right). \end{array}$$

These equations imply that the difference between the expression using the true state and the one using the estimated state converges to zero, implying that the two tests accept and reject jointly asymptotically under the null hypothesis.
